# Supplementary material for: Quantitative modelling of amino acid transport and homeostasis in mammalian cells
Source: Nat Commun. 2021 Sep 6;12:5282. doi: 10.1038/s41467-021-25563-x (PMC8421413; doi:10.1038/s41467-021-25563-x)
Supplement: Supplementary file 1 — Supplemental Information [file 41467_2021_25563_MOESM1_ESM.pdf]

Supplementary Information

Quantitative modelling of amino acid transport and homeostasis in mammalian cells

Gregory Gauthier-Coles[1], Jade Vennitti[1], Zhiduo Zhang[2], William C. Comb[3], Shuran Xing [3], Kiran Javed[1], Angelika Broer[1], Stefan Broer*[1]

Fig.S1

U87-MG Transporter expression

U87-MG		Database	mRNA	Protein	Evaluation
EAAT3	SLC1A1	-0.36	0.33	nd	-
EAAT2	SLC1A2	-1.4	0.08	nd	-
EAAT1	SLC1A3	3.1	0.56	0.11	+
ASCT1	SLC1A4	3.5	0.78	0.25	++
ASCT2	SLC1A5	5.4	1.08	1.13	+++
EAAT4	SLC1A6	-1.5	0	nd	-
EAAT5	SLC1A7	-0.1	0	nd	-
rbat	SLC3A1	0.33	0	nd	-
4F2hc	SLC3A2	4.5	0.62	nd	+++
GLYT2	SLC6A5	-0.5	0.72	0.03	+
PROT	SLC6A7	0.4	0	nd	-
GLYT1	SLC6A9	-0.4	0	nd	-
ATB0+	SLC6A14	-1.1	0	nd	-
B0AT2	SLC6A15	2.8	0.5	0.99	+++
NTT5	SLC6A16	-0.7	0	nd	-
NTT4	SLC6A17	0.8	0.02	nd	-
B0AT1	SLC6A19	0	0	nd	-
SIT	SLC6A20	-1.3	0	nd	-
CAT1	SLC7A1	0.9	0.79	1.48	+++
CAT2	SLC7A2	-1	0.1	nd	-
CAT3	SLC7A3	-1	0	nd	-
CAT4	SLC7A4	-0.7	0.3	nd	-
LAT1	SLC7A5	5.7	0.91	0.54	+++
y+LAT2	SLC7A6	1.9	0.16	0.79	+++
y+LAT1	SLC7A7	-0.1	0	nd	-
LAT2	SLC7A8	0.4	0.52	0.07	+
b0+AT	SLC7A9	0.2	0	nd	-
asc1	SLC7A10	nd	0	nd	-
xCT	SLC7A11	1.5	0.9	0.65	+++
TAT1	SLC16A10	0.1	0.03	nd	-
PAT1	SLC36A1	-0.9	0.7	nd	-
PAT2	SLC36A2	nd	0.06	nd	-
PAT3	SLC36A3	nd	0.03	nd	-
PAT4	SLC36A4	2.8	0.58	nd	++
SNAT1	SLC38A1	6.5	1.05	1.69	+++
SNAT2	SLC38A2	6.4	1.14	0.01	inducible
SNAT3	SLC38A3	-0.7	0	nd	-
SNAT4	SLC38A4	-1.3	0.14	0.12	+
SNAT5	SLC38A5	-0.1	0	0.63	++
LAT3	SLC43A1	0.3	0.18	0.02	-
LAT4	SLC43A2	-0.3	0.25	nd	-

A549 Transporter expression

A549		Database	mRNA	Protein	Evaluation
EAAT3	SLC1A1	-0.5	0.53872	0.00	-
EAAT2	SLC1A2	-1.6	0	nd	-
EAAT1	SLC1A3	-1.3	0	nd	-
ASCT1	SLC1A4	3.5	1.55816	0.00	-
ASCT2	SLC1A5	5.7	1.73847	1.62	+++
EAAT4	SLC1A6	-1.4	0	nd	-
EAAT5	SLC1A7	-0.4	0	nd	-
rbat	SLC3A1	3.06	0.10848	nd	-
4F2hc	SLC3A2	6.7	0.94904	nd	+++
GLYT2	SLC6A5	-0.7	0.9382	0.00	-
PROT	SLC6A7	0.2	0	nd	-
GLYT1	SLC6A9	-0.5	0	nd	-
ATB0+	SLC6A14	-1.4	0	nd	-
B0AT2	SLC6A15	0.5	0	nd	-
NTT5	SLC6A16	0.22	0.06	nd	-
NTT4	SLC6A17	0.4	0.18499	nd	-
B0AT1	SLC6A19	0.2	0	nd	-
SIT	SLC6A20	-1.3	0	nd	-
CAT1	SLC7A1	-0.6	1.39068	0.01	-
CAT2	SLC7A2	-0.7	0.1327	nd	-
CAT3	SLC7A3	-1	0.01713	nd	-
CAT4	SLC7A4	-0.7	0.0256	nd	-
LAT1	SLC7A5	8.5	1.92245	0.77	+++
y+LAT2	SLC7A6	2.9	0.53168	0.34	++
y+LAT1	SLC7A7	4.4	0.09901	nd	-
LAT2	SLC7A8	0.3	1.29083	0.04	+
b0+AT	SLC7A9	0.6	0	nd	-
asc1	SLC7A10		0	nd	-
xCT	SLC7A11	4.7	1.5445	0.64	+++
TAT1	SLC16A10	-0.3	0.0048	nd	-
PAT1	SLC36A1	-0.9	0.28336	nd	-
PAT2	SLC36A2		0.02102	nd	-
PAT3	SLC36A3		0.02687	nd	-
PAT4	SLC36A4	4	0.60582	nd	-
SNAT1	SLC38A1	6.6	0.9867	3.56	+++
SNAT2	SLC38A2	6.7	0.855	0.00	inducible
SNAT3	SLC38A3	-0.5	0	nd	-
SNAT4	SLC38A4	-1.4	0	0.03	-
SNAT5	SLC38A5	-0.6	0.028	0.26	+
LAT3	SLC43A1	0.3	0.24753	0.75	++
LAT4	SLC43A2	0.6	0.73334	nd	+

Fig. S1 Evaluation of transporter expression in U87-MG glioma and A549 lung carcinoma cells**.** The mRNA expression levels were compiled from Oncomine (log2 normalised mRNA expression) and analysed by RT-PCR (normalised to clathrin intensity). Red indicates high level of expression, blue indicates low levels of expression. High levels of mRNA expression were correlated to western blotting (normalised to Na^{+}/K^{+}-ATPase intensity e= 3). Overall evaluation was based on the combined dataset. +++ high activity expected, ++ activity expected to be readily detectable, + marginal activity, - no activity expected. The activity of SNAT2 is inducible and may not be detectable in cells exposed to freshly media.

Fig.S2

U87-MG   A549

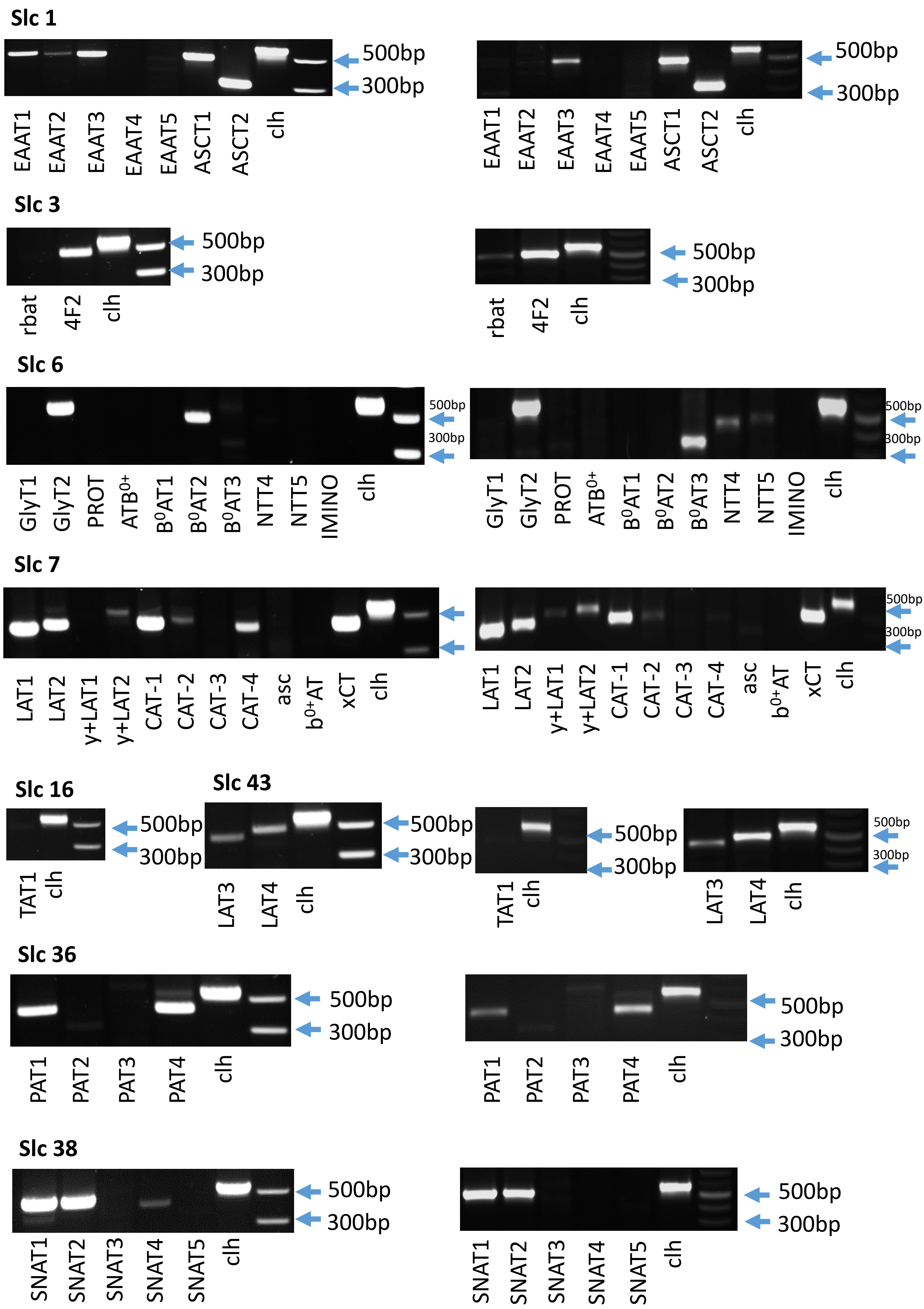

Fig. S2: Analysis of transporter expression by RT-PCR. For reverse transcription 2 µg of total RNA isolated from U87-MG cells or A549 cells was used in a reaction volume of 20 µL. One µl of the cDNA sample was used for a 30 cycle PCR reaction. Predicted fragment sizes are listed in the methods section (representative gel from e=3 experiments).

U87-MG

Fig.S3

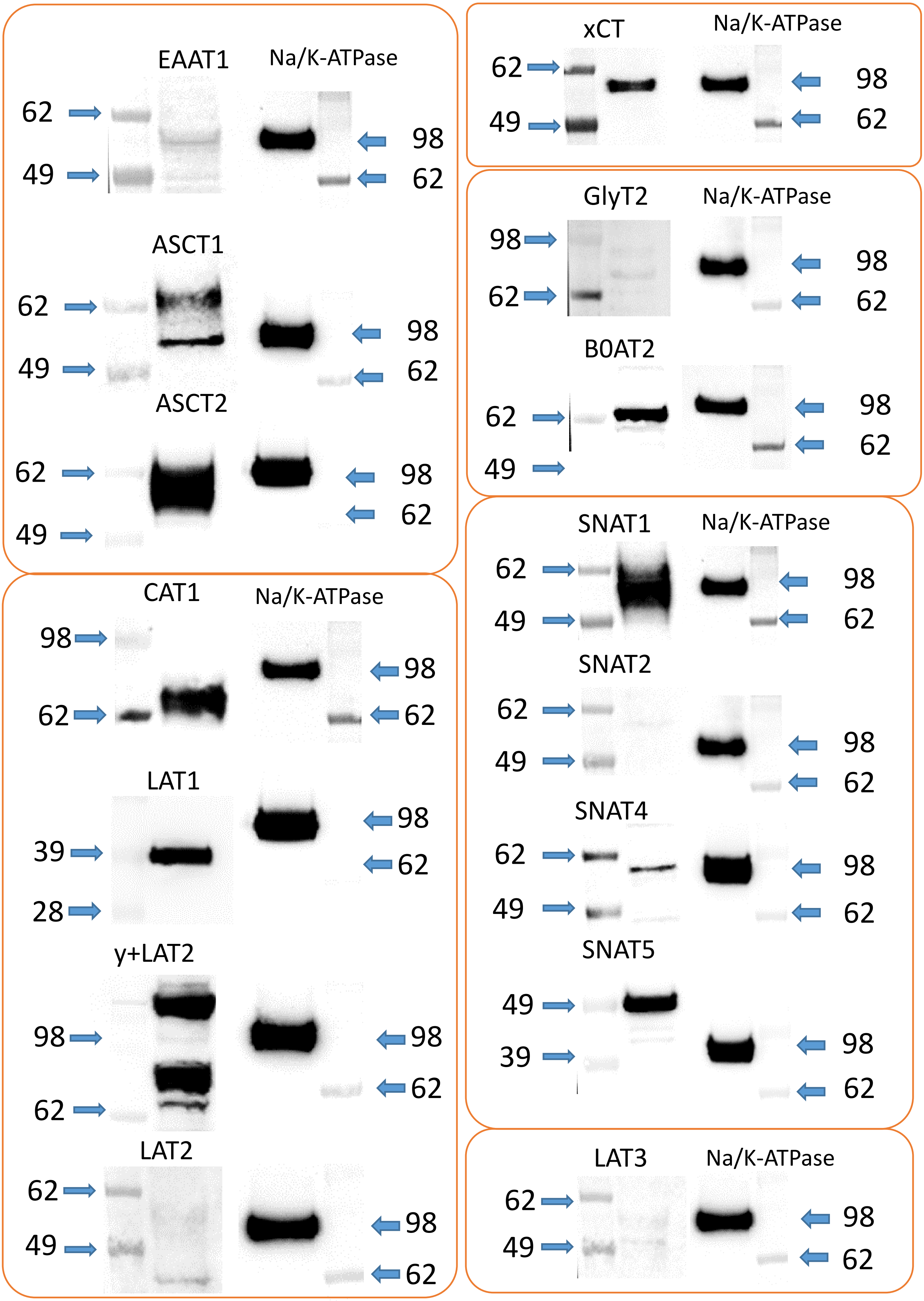
EAAT1
Na/K-ATPase
62
49
98
62
ASCT1
62
49
98
62
ASCT2
62
49
98
62
CAT1
Na/K-ATPase
98
62
98
62
LAT1
39
28
98
62
y+LAT2
98
62
98
62
LAT2
62
49
98
62
xCT
Na/K-ATPase
62
49
98
62
GlyT2
Na/K-ATPase
98
62
98
62
B0AT2
62
49
98
62
SNAT1
Na/K-ATPase
62
49
98
62
SNAT2
62
49
98
62
SNAT4
62
49
98
62
SNAT5
49
39
98
62
LAT3
Na/K-ATPase
62
49
98
62

Fig S3. Surface expression of amino acid transporters in U87-MG cells. Surface biotinylation was performed before detection of amino acid transporters by western blotting (Representative blot from e=3 experiments). Antibodies were used as listed in table S12. For comparison Na^+/K^+-ATPase was detected on the same blot after stripping. Molecular weight of marker proteins is given in kDa.

A549

Fig.S4

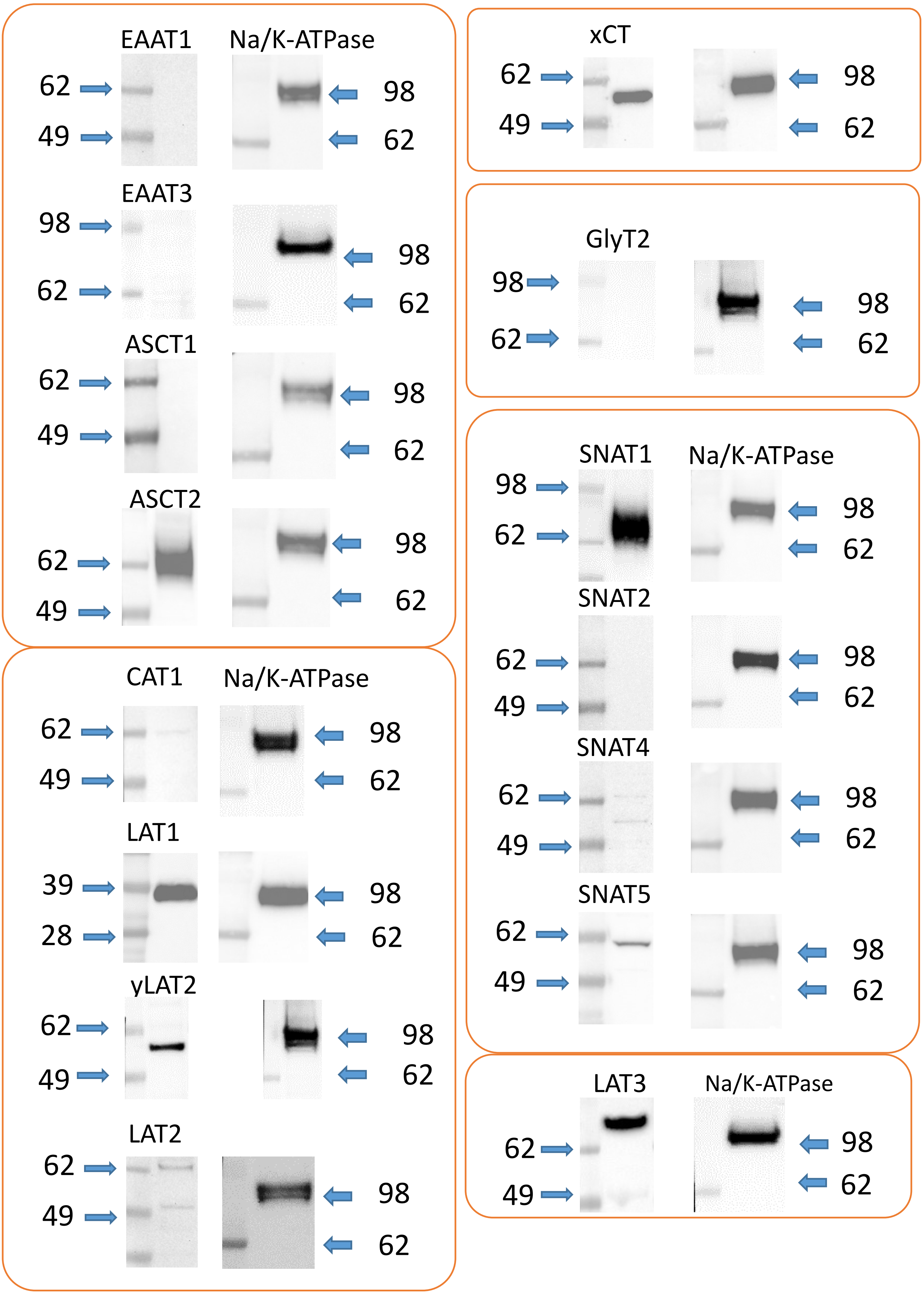
EAAT1
Na/K-ATPase
62
49
98
62
EAAT3
98
62
98
62
ASCT1
62
49
98
62
ASCT2
62
49
98
62
CAT1
Na/K-ATPase
62
49
98
62
LAT1
39
28
98
62
yLAT2
62
49
98
62
LAT2
62
49
98
62
xCT
62
49
98
62
GlyT2
98
62
98
62
SNAT1
Na/K-ATPase
98
62
98
62
SNAT2
62
49
98
62
SNAT4
62
49
98
62
SNAT5
62
49
98
62
LAT3
Na/K-ATPase
62
49
98
62

Fig S4. Surface expression of amino acid transporters in A549 cells. Surface biotinylation was performed before detection of amino acid transporters by western blotting (Representative blot from e=3 experiments). Antibodies were used as listed in table S12. For comparison Na^{+}/K^{+}-ATPase was detected on the same blot after stripping. Molecular weight of marker proteins is given in kDa.

a

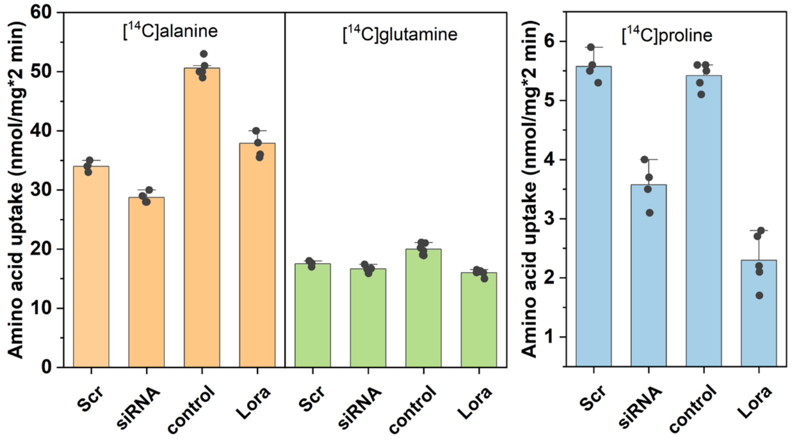

b

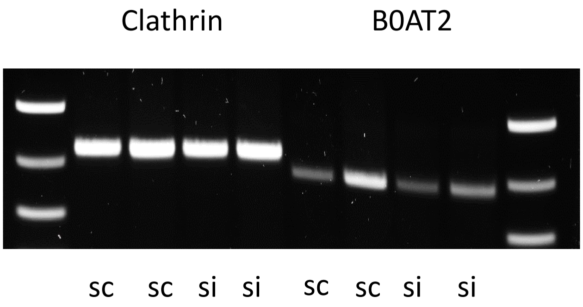

d u d u d u d u

d: diluted 1:3 RT reaction
u: undiluted RT reaction
sc: scrambled
si: B^0AT2 RNAi

Fig. S5: Contribution by B^0AT2 to amino acid transport in U87-MG cells. (a) Alanine, glutamine and proline transport was measured in control cells and in cells where B^0AT2 was silenced by RNAi. For comparison, inhibition by the published B^0AT2 inhibitor loratadine (lora) is shown. Error bars indicate standard deviation. Numbers of biologically independent samples (n) from left to right n=3,4,5,5,3,4,6,6,4,4,5,5; e=3. (b) Silencing of B^0AT2 mRNA (si), using 1:3 diluted and undiluted reverse transcriptase reactions to amplify B^0AT2 or clathrin (control) mRNA. Scrambled RNA (sc) was used as a control. Representative gel from e=3 experiments.

Fig.S6

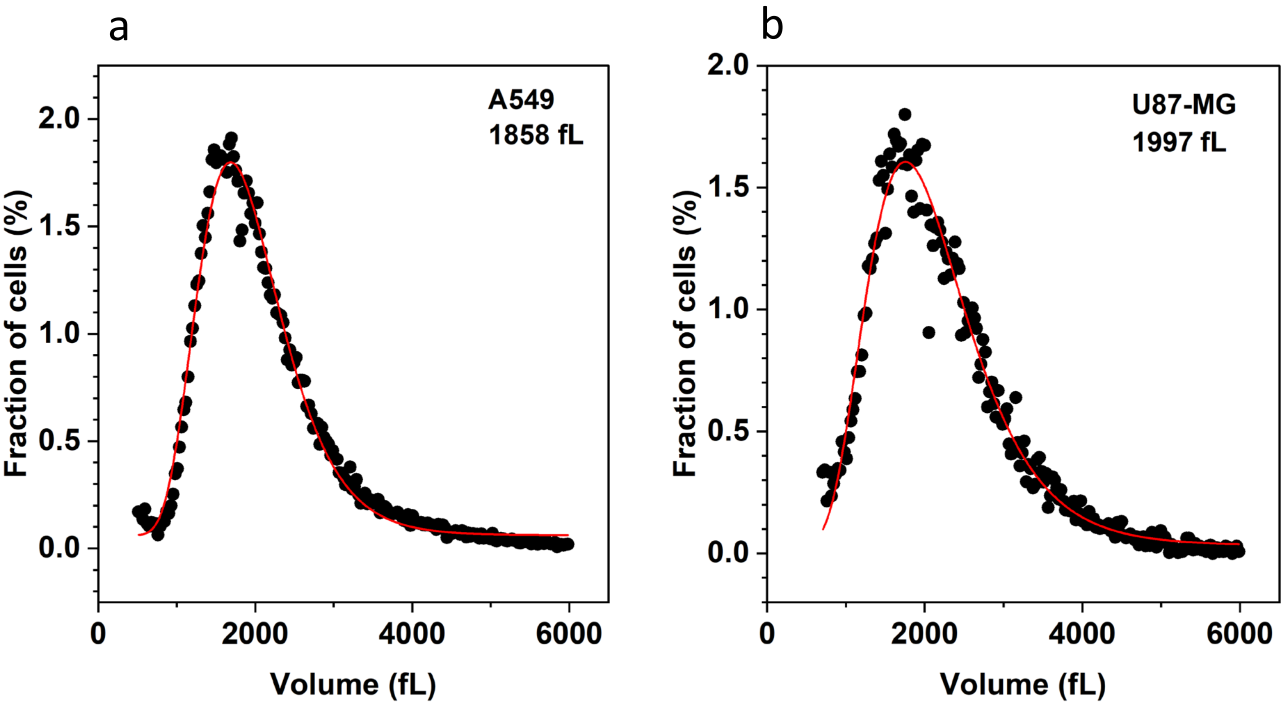

Fig. S6: Volume analysis of cell lines. Cell lines A549 (A) and U87-MG (B) were dislodged by trypsin treatment and dispersed by passage through 70 µm sieve. Cell size was measured by a Multisizer 4 Coulter counter.

Fig.S7

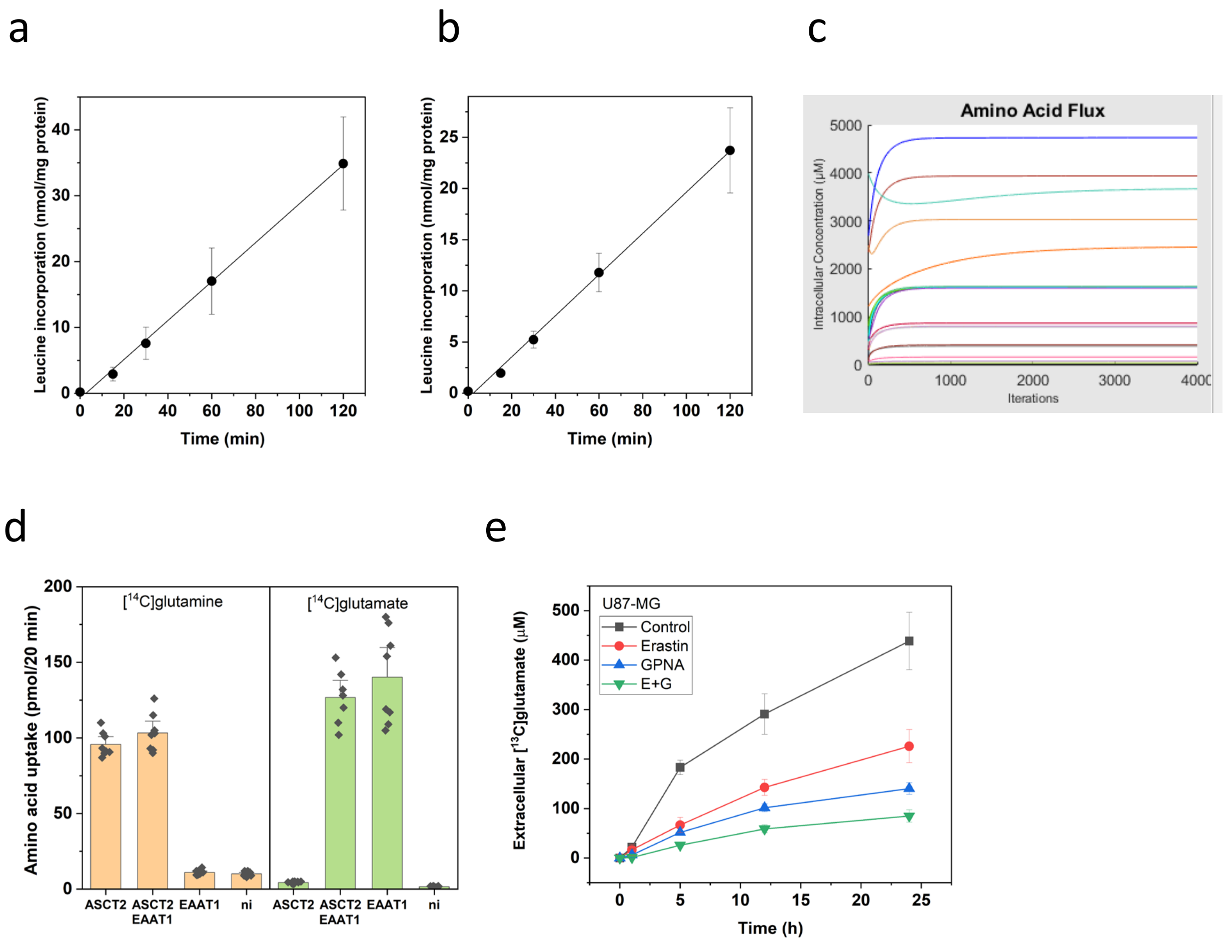

Fig. S7: Quantification and verification of modelling parameters. a,b) Incorporation of leucine into protein over time in A549 (a) and U87-MG (b) cells. n=3, e=3. c) Snapshot of JDFC demonstrating a stable equilibrium of cytosolic amino acid concentrations starting from low initial concentrations. d) Activity of transporters in *Xenopus laevis* oocytes was confirmed by uptake of 100 µM [^{14}C]glutamate and 100 µM [^{14}C]glutamine (n=8,8,11,11,11,12,7,8,11; e=1). The 95% confidence intervals are shown. e) Efflux of glutamate in U87-MG cells was measured by feeding cells with [^{13}C]glutamine in BME medium supplemented with non-essential amino acids and detecting M+5 glutamate in the media over time. To discriminate between glutamate efflux pathways, xCT inhibitor erastin (10 µM) and ASCT2 inhibitor GPNA (10 mM) were used. Data are shown as mean ± STD (n=15, e=3).

Fig.S8

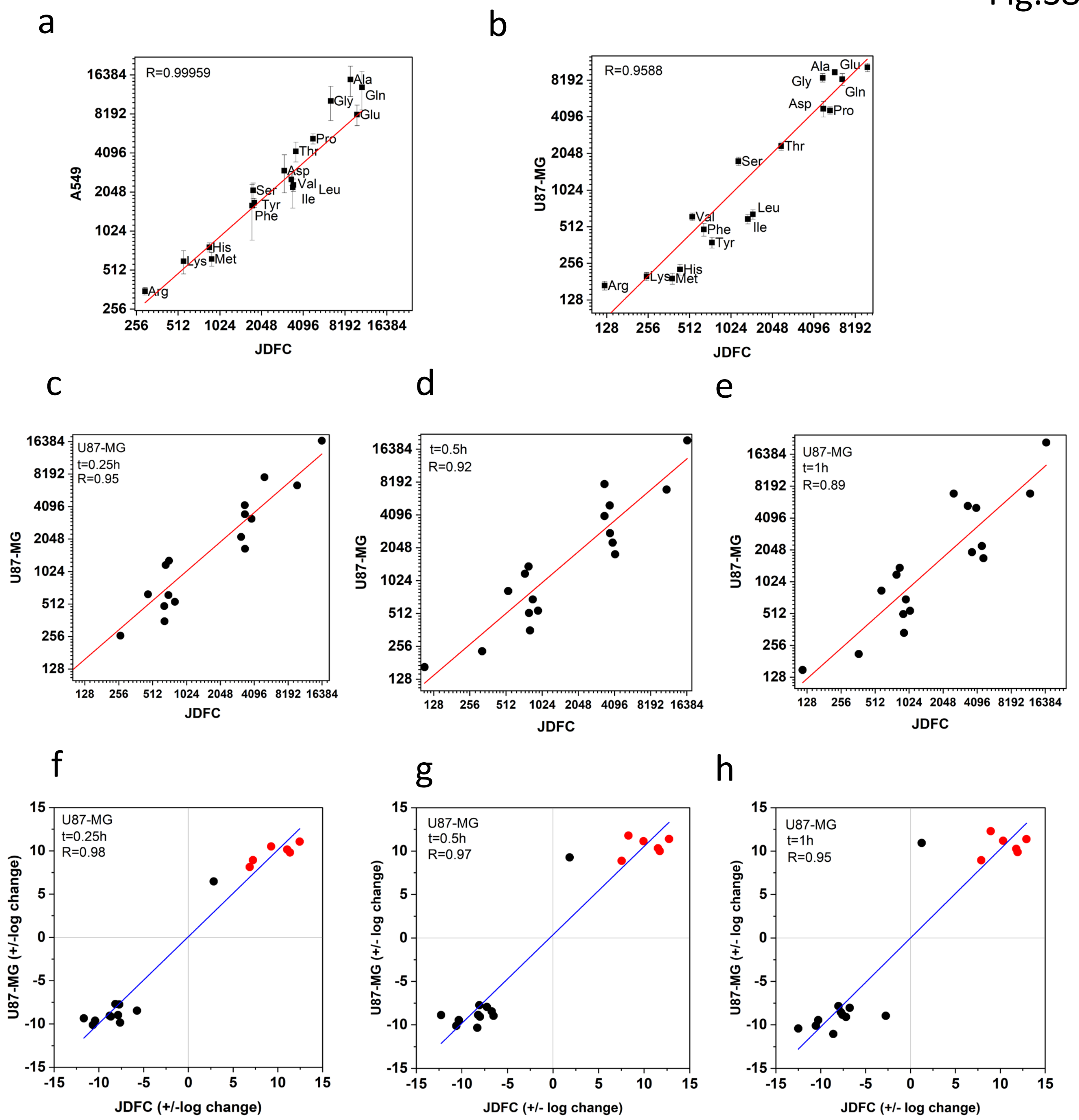

Fig. S8: Modelling of amino acid transport. a,b) Correlation between experimental and simulated amino acid concentrations in A549 cells (a) and U87-MG cells (b) Error bars indicate the mean ± STD. (c-h) U87-MG cells were incubated with an amino acid mixture of all proteinogenic amino acids (100 µM each). Five amino acids were spiked at a concentration of 500 µM. Cytosolic amino acid concentrations were determined by LC-MS before addition of amino acid mixtures and after 15, 30 and 60 min. The correlation between experimental data and simulation were analysed using absolute concentrations (c,d,e) and relative changes (log2 change) for each time point (f,g,h) plotted in a quadrant chart. Spiked amino acids are shown as red dots in panels f, g, h. Mean concentrations of n=3 experiments are shown.

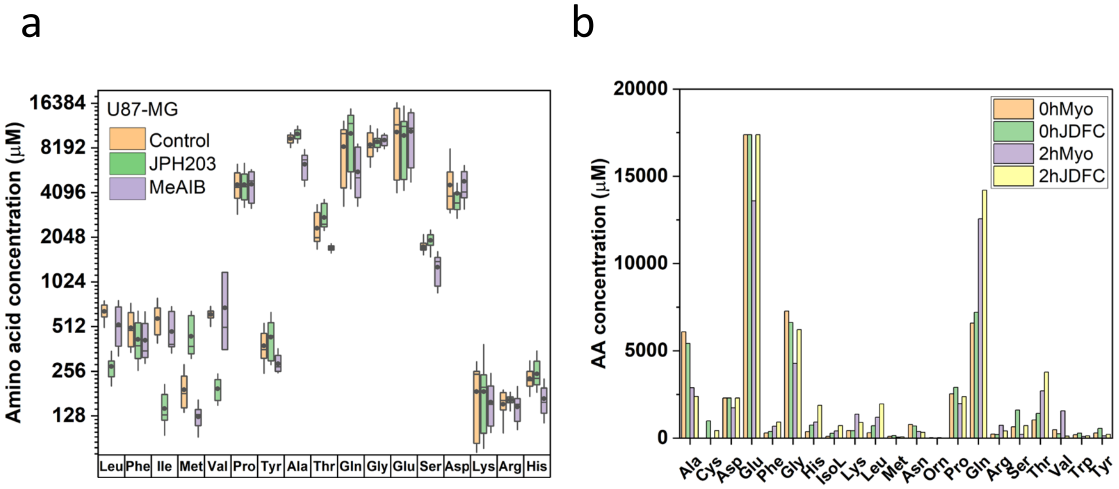

Fig. S9 Disturbing amino acid homeostasis. a) Effect of amino acid transport inhibitors JPH203 and MeAIB on cytosolic amino acid concentrations in U87-MG cells after 1h incubation (n=15, e=3, data are shown as box-whisker plot showing range, 25-75% area, median and mean. b) Experimental and simulated cytosolic amino acid concentrations in human myotubes grown in sarcopenia-inducing medium after incubation with AXA2678 for 2h. Mean concentration of 2 independent experiments are shown). Sample amino acid concentrations were multiplied x113 based on morphometric volume measurements in parallel experiments..

Table S1: Experimental conditions to isolate amino acid transport activities.

Transport Substrate	Ion	Inhibitor/competitor (conc.)	Detectable Transporters
Arginine.	Na+	None	ATB$^{0,+}$ / CAT1,2,3 / y+LAT1,2 / b$^{0,+}$AT
100 µM.	Na+	Me-DL-Trp (2.5 mM, ATB$^{0,+}$)	/ CAT1,2,3 / y+LAT1,2 / b$^{0,+}$AT
6 minutes.	Na+	NEM (0.4 mM pre-treated)	ATB$^{0,+}$ / / y+LAT1,2 / b$^{0,+}$AT
	Na+	Leucine (1 mM)	/ CAT1,2,3 / /
	NMDG	Leucine (1 mM)	/ CAT1,2,3 / y+LAT1,2 /
	Na+	Arginine (10 mM)	None
Glutamate	Na+	None	EAAT1,2,3,4,5 / xCT
25 µM.	NMDG	None	/ xCT
6 minutes.	Na+	Cysteine (10 mM)	EAAT1,2, ,4,5 / xCT
	Na+	Dihydrokainate (200 µM, EAAT2)	EAAT1, ,3,4,5 / xCT
	Na+	UCPH101 (10 µM, EAAT1)	EAAT ,2,3,4,5 / xCT
	Na+	TBOA (0.1 mM and 0.5 mM)	/ xCT
	Na+	Sulfasalazine (0.5 mM, xCT)	EAAT1,2,3,4,5 /
	Na+	Erastin (10 µM)	EAAT1,2,3,4,5 /
	Na+	Glutamate (10 mM)	None
Leucine	Na+	None	ATB$^{0,+}$ / B^{0}AT1,2,NTT4 / LAT1,2,3,4 / y+LAT1,2 / SNAT2
100 µM.	NMDG	None	/ LAT1,2,3,4 /
2 minutes.	Na+	Me-DL-Trp (2.5mM, ATB$^{0,+}$)	/ B^{0}AT1,2,NTT4 / LAT1,2,3,4 / y+LAT1,2 / SNAT2
	Na+	Cinromide (30 µM, B^{0}AT1)	ATB$^{0,+}$ / B^{0}AT 2,NTT4 / LAT1,2,3,4 / y+LAT1,2 / SNAT2
	Na+	Loratadine (50 µM, B^{0}AT2)	ATB$^{0,+}$ / B^{0}AT1 ,NTT4 / LAT1,2,3,4 / y+LAT1,2 / SNAT2
	Na+	BCH (10 mM)	/ NTT4 / / y+LAT1,2 / SNAT2
	Na+	JPH203 (3 µM, LAT1)	ATB$^{0,+}$ / B^{0}AT1,2,NTT4 / LAT 2,3,4 / y+LAT1,2 / SNAT2
	Na+	Arginine (1 mM)	/ B^{0}AT1,2,NTT4 / LAT1,2,3,4 / / SNAT2
	Na+	MeAIB (10 mM)	ATB$^{0,+}$ / B^{0}AT1,2,NTT4 / LAT1,2,3,4 / y+LAT1,2 /
	Na+	Leucine (30 mM)	None
Phenylalanine	Na+	None	ATB$^{0,+}$ / B^{0}AT1 / LAT1,2,3,4 / y+LAT1,2 / TAT1
100 µM	NMDG	None	/ LAT1,2,3,4 / / TAT1
2 minutes	Na+	NEM (0.4 mM pretreated)	ATB$^{0,+}$ / B^{0}AT1 / LAT1,2, / y+LAT1,2 / TAT1
	Na+	Me-DL-Trp (2.5 mM, ATB$^{0,+}$)	/ B^{0}AT1 / LAT1,2,3,4 / y+LAT1,2 / TAT1
	Na+	Cinromide (3 µM, B^{0}AT1)	ATB$^{0,+}$ / / LAT1,2,3,4 / y+LAT1,2 / TAT1
	Na+	JPH203 (3 µM, LAT1)	ATB$^{0,+}$ / B^{0}AT1/ LAT 2,3,4 / y+LAT1,2 / TAT1
	Na+	Arginine (1 mM)	/ B^{0}AT1/ LAT1,2,3,4 / / TAT1
	NMDG	BCH (10 mM)	ATB$^{0,+}$ / B^{0}AT1/ / y+LAT1,2 / TAT1
	+Na	Phenylalanine (30 mM)	None
Alanine	Na+	None	ASCT1,2 / ATB$^{0,+}$ / B^{0}AT1,2 / asc-1 / LAT2 / PAT1,2 / SNAT1,2,4,5
300 µM	NMDG	None	/ / / asc-1 / LAT2 / PAT1,2 /
2 minutes	Na+	GPNA (3 mM)	ASCT1, / / B^{0}AT1,2 / asc-1 / LAT2 / PAT1,2 / SNAT ,4,5
	Na+	Me-DL-Trp (2.5 mM, ATB$^{0,+}$)	ASCT1,2 / / B^{0}AT1,2 / asc-1 / LAT2 / PAT1,2 / SNAT1,2,4,5
	Na+	Cinromide (3 µM, B^{0}AT1)	ASCT1,2 / ATB$^{0,+}$ / B^{0}AT ,2 / asc-1 / LAT2 / PAT1,2 / SNAT1,2,4,5
	Na+	BMS-466442 (2uM, asc-1)	ASCT1,2 / ATB$^{0,+}$ / B^{0}AT1,2 / / LAT2 / PAT1,2 / SNAT1,2,4,5
	Na+	Loratadine (50 µM)	ASCT1,2 / ATB$^{0,+}$ / B^{0}AT1 / asc-1 / LAT2 / PAT1,2 / SNAT1,2,4,5
	NMDG	BCH (10 mM)	ASCT1,2/ / / asc-1 / / PAT1,2 / SNAT1,2,4,5
	Na+	MeAIB (10 mM)	ASCT1,2 / ATB$^{0,+}$ / B^{0}AT1,2 / asc-1 / LAT2 / / SNAT 5
	Na+	GABA (10 mM)	ASCT1,2 / ATB$^{0,+}$ / B^{0}AT1,2 / asc-1 / LAT2 / PAT ,2 / SNAT1,2,4,5
	Na+	Betaine (10 mM)	ASCT1,2 / ATB$^{0,+}$ / B^{0}AT1,2 / asc-1 / LAT2 / PAT1,2 / SNAT1, ,4,5
	Na+	Alanine (30 mM)	None
Glutamine	Na+	None	ASCT2 / ATB$^{0,+}$ / B^{0}AT1,2 / LAT1,2 / y+LAT1,2/ SNAT1,2,3,4,5
100 µM	NMDG	None	/ / / LAT1,2 / /
2 minutes.	Na+	Me-DL-Trp (2.5 mM, ATB$^{0,+}$)	ASCT2 / / B^{0}AT1 / LAT1,2 / y+LAT1,2/ SNAT1,2,3,4,5
	Na+	Cinromide (3 µM, B^{0}AT1)	ASCT2 / ATB$^{0,+}$ / / LAT1,2 / y+LAT1,2/ SNAT1,2,3,4,5
	Na+	JPH203 (3 µM, LAT1)	ASCT2 / ATB$^{0,+}$ / B^{0}AT1 / LAT 2 / y+LAT1,2/ SNAT1,2,3,4,5
	Na+	BCH (10 mM)	ASCT2 / ATB$^{0,+}$ / / / y+LAT1,2/ SNAT1,2,3,4,5
	Na+	Arginine (10 mM)	ASCT2 / / B^{0}AT1 / LAT1,2 / / SNAT1,2,3, ,5
	Li+	None	/ / / LAT1,2 / y+LAT1,2/ SNAT 3, ,5
	Na+	MeAIB (10 mM)	ASCT2/ ATB$^{0,+}$ / B^{0}AT1 / LAT1,2 / y+LAT1,2/ SNAT ,3, ,5
	Na+	Betaine (10 mM)	ASCT2 / ATB$^{0,+}$ / B^{0}AT1 /LAT1,2 / y+LAT1,2/ SNAT1, ,3,4,5
	NMDG	BCH (10mM)	ASCT2 / ATB$^{0,+}$ / B^{0}AT1 / / y+LAT1,2/ SNAT1,2,3,4,5
	Na+	Glutamine (10 mM)	None
Glycine	Na+	None	ASCT2/ GlyT1,2 / ATB$^{0,+}$ / B^{0}AT1 / asc-1 / LAT2 / PAT1,2 / SNAT1,2,4,5
100 µM	NMDG	None	/ / / / asc-1 / LAT2 / PAT1,2 /
6 minutes.	Na+	ALX5407(1 µM, GlyT1)	ASCT2 / GlyT ,2 / ATB$^{0,+}$ / B^{0}AT1 / asc-1 / LAT2 / PAT1,2 / SNAT1,2,4,5
	Na+	ORG25543 (0.2 µM, GlyT2)	ASCT2/ GlyT1, / ATB$^{0,+}$ / B^{0}AT1 / asc-1 / LAT2 / PAT1,2 / SNAT1,2,4,5
	Na+	Me-DL-Trp (2.5 mM, ATB$^{0,+}$)	ASCT2/ GlyT1,2 / / B^{0}AT1 / asc-1 / LAT2 / PAT1,2 / SNAT1,2,4,5
	Na+	Cinromide (3 µM, B^{0}AT1)	ASCT2/ GlyT1,2 / ATB$^{0,+}$ / / asc-1 / LAT2 / PAT1,2 / SNAT1,2,4,5
	Na+	BMS-466442 (2 µM, asc-1)	ASCT2/ GlyT1,2 / ATB$^{0,+}$ / B^{0}AT1 / / LAT2 / PAT1,2 / SNAT1,2,4,5
	NMDG	BCH (10 mM)	ASCT2/ GlyT1,2 / / / asc-1 / / PAT1,2 / SNAT1,2,4,5
	Na+	MeAIB (10 mM)	ASCT2/ GlyT1,2 / ATB$^{0,+}$ / B^{0}AT1 / asc-1 / LAT2 / / SNAT 5
	Na+	GABA (15 mM)	ASCT2/ GlyT1,2 / ATB$^{0,+}$ / B^{0}AT1 / asc-1 / LAT2 / PAT ,2 / SNAT1,2,4,5
	Na+	Betaine (10 mM)	ASCT2/ GlyT1,2 / ATB$^{0,+}$ / B^{0}AT1 / asc-1 / LAT2 / PAT1,2 / SNAT1, ,4,5
	Na+	Glycine (30 mM)	None
Proline	Na+	None	ASCT1 / PROT / B^{0}AT1,2 / SIT / asc-1 / PAT1,2,4 / SNAT1,2,4,5

100 µM<br>6 minutes.	NMDG	None	/ / / / PAT1,2,4 /
	Na+	LP-403812 (10 µM, PROT)	ASCT1 / / B^0AT1,2 / SIT / PAT1,2,4 / SNAT1,2,4,5
	Na+	Cinromide (3 µM, B^0AT1)	ASCT1 / PROT / B^0AT ,2 / SIT / PAT1,2,4 / SNAT1,2,4,5
	Na+	Loratadine (50 µM, B^0AT2)	ASCT1 / PROT / B^0AT1, / SIT / PAT1,2,4 / SNAT1,2,4,5
	Na+	Tryptophan (1 mM)	ASCT1 / PROT / B^0AT1,2 / SIT / PAT1,2, / SNAT1,2,4,5
	Na+	L-trans-4-hydroxyproline (200 µM)	/ PROT / B^0AT1,2 / SIT / PAT1,2,4 / SNAT1,2,4,5
	Na+	GABA (15 mM)	ASCT1 / PROT / B^0AT1,2 / SIT / PAT ,2,4 / SNAT1,2,4,5
	Na+	MeAIB (10 mM)	ASCT1 / PROT / B^0AT1,2 / / PAT ,4 / SNAT ,5
	Na+	Proline (30 mM)	None

Table S2: Amino acid transporters and their mechanisms. Transporters are listed by solute carrier family number and common name. The corresponding transport activity in cultured cells is shown in column "Activity". Substrates are shown in one-letter code or as groups with AA^0 indicating neutral amino acids, AA^+ indicating cationic amino acids, O, ornithine, Cit, citrulline, Cn, cysteine. S: symport, A: antiport, subcellular location is shown as PM, plasma membrane; V, vesicular; M, mitochondria; Ly, lysosome.

SLC	Acronym	Substrates	Activity	Mechanism	Loc
SLC1A1	EAAT3	D,E	System X_{AG}	S:3Na+/1H+A:1K+	PM
SLC1A2	EAAT2	D,E	System X_{AG}	S:3Na+/1H+A:1K+	PM
SLC1A3	EAAT1	D,E	System X_{AG}	S:3Na+/1H+A:1K+	PM
SLC1A4	ASCT1	A,S,C	System ASC	Antiporter	PM
SLC1A5	ASCT2	A,S,C,T,Q,N	System ASC	Antiporter	PM
SLC1A6	EAAT4	D,E	System X_{AG}	S:3Na+/1H+A:1K+	PM
SLC1A7	EAAT5	D,E	System X_{AG}	S:3Na+/1H+A:1K+	PM
SLC3A1	rBAT	Trafficking	Heavy chains of		PM
SLC3A2	4F2hc	subunits	Heteromeric AA		PM
SLC6A5	GlyT2	G	System Gly	S:3Na+/1Cl-	PM
SLC6A7	PROT	P	Proline transporter	S:2Na+/1Cl-	V/PM
SLC6A9	GlyT1	G	System Gly	S:2Na+/1Cl-	PM
SLC6A14	$ATB^{0,+}$	All AA^0, AA^+	System $B^{0,+}$	S:2Na+/1Cl-	PM
SLC6A15	B^0AT2	P,L,V,I,M	SystemB^0	S:1Na+	PM
SLC6A17	NTT4/B^0AT3	L,M,P,C,A,Q,S,H,G	SystemB^0	S:2Na+/1Cl-	PM/V
SLC6A18	XT2/B^0AT3	G,A	System Gly	S:2Na+/1Cl-	PM
SLC6A19	B^0AT1	All AA^0	System B^0	S:1Na+	PM
SLC6A20	SIT1	P (F,L)	System IMINO	S:2Na+/1Cl-	PM
SLC7A1	CAT-1	K,R,O	System y+	Uniporter	PM
SLC7A2	CAT-2	K,R,O	System y+	Uniporter	PM
SLC7A3	CAT-3	K,R,O	System y+	Uniporter	PM
SLC7A4	CAT-4	unknown	unknown	unknown	Ly
SLC7A5	LAT1/4F2hc	H,M,L,I,V,F,Y,W	System L	Antiporter	PM
SLC7A6	y+LAT2/4F2hc	K,R,Q,H,M,L	System y+L	S:AA0-Na+A:AA+	PM
SLC7A7	y+LAT1/4F2hc	K,R,Q,H,M,L,A,C	System y+L	S:AA0-Na+A:AA+	PM
SLC7A8	LAT2/4F2hc	All AA^0 (-P)	System L	Antiporter	PM
SLC7A9	b0,+AT/rBAT	R,K,O,Cn	System $b^{0,+}$	Antiporter	PM
SLC7A10	Asc-1/4F2hc	G,A,S,C,T	System asc	Antiporter	PM
SLC7A11	xCT/4F2hc	E,Cn	Sytem x^-_c	Antiporter	PM
SLC7A12	Asc-2	G,A,S,C,T	System asc	Antiporter	PM
SLC7A13	AGT1/rBAT	Cn	Cystine transporter	Antiporter	PM
SLC7A14		K,R,O	System c	Uniporter	Ly
SLC16A10	TAT1	W,Y,F	System T	Uniporter	PM
SLC17A6	VGLUT2	E	Vesicular glutamate transporter	Uniporter	V
SLC17A7	VGLUT1	E	Vesicular glutamate transporter	Uniporter	V
SLC17A8	VGLUT3	E	Vesicular glutamate transporter	Uniporter	V
SLC25A2	ORC2	K,R,H,O,Cit	Orn/Cit carrier	Antiporter	M
SLC25A12	AGC1	D,E	Asp/Glu carrier	Antiporter	M
SLC25A13	AGC2	D,E	Asp/Glu carrier	Antiporter	M
SLC25A15	ORC1	K,R,H,O,Cit	Orn/Cit carrier	Antiporter	M
SLC25A18	GC2	E	Glu carrier	A:OH-	M
SLC25A22	GC1	E	Glu carrier	A:OH-	M
SLC32A1	VIAAT	G,GABA	Vesicular Gly/GABAtransporter	A:H+	V

SLC36A1	PAT1	G,P,A	Proton-amino acid transporter	S:H+	PM,Ly
SLC36A2	PAT2	G,P,A	Proton-amino acid transporter	S:H+	PM
SLC36A3	PAT3	unknown	unknown	unknown	PM
SLC36A4	PAT4	P,W	Amino acid sensor	unknown	PM,L
SLC38A1	SNAT1	G,A,N,C,Q,H,M	System A	S:Na+	PM
SLC38A2	SNAT2	G,P,A,S,C,Q,N,H,M	System A	S:Na+	PM
SLC38A3	SNAT3	Q,N,H	System N	S:Na+A:H+	PM
SLC38A4	SNAT4	G,A,S,C,Q,N,M	System A	S:Na+	PM
SLC38A5	SNAT5	Q,N,H,A	System N	S:Na+A:H+	PM
SLC38A6	SNAT6	Unknown	Unknown	unknown	
SLC38A7	SNAT7	Q,N,H,A,S,D	Unknown	S:Na+	Ly
SLC38A8	SNAT8	Q,A,R,H,D	System A	S:Na+	PM
SLC38A9	SNAT9	Q,R,N	Lysosomal transporter	S:Na+	Ly
SLC38A10	SNAT10	Unknown	Unknown	Unknown	
SLC38A11	SNAT11	Unknown	Unknown	Unknown	
SLC43A1	LAT3	L,I,M,F,V	System L	Uniporter	PM
SLC43A2	LAT4	L,I,M,F,V	System L	Uniporter	PM

Table S3: Substrate K_M-Values of known amino acid transporters. The table shows a curated list of K_M-values, which in the opinion of the authors represent the most reliable experimental values. Many studies have not determined the Km of all possible substrates, in which case an estimate (est) was made based on physical relation to known substrate Km-values and general trends of Km-values and competitive inhibition observed for a particular transporter. All K_M –values are shown in µM.

**SLC1 family**	
***SLC1A1* (EAAT3)**	[1, 2, 3, 4]
Glutamate	62±8
Aspartate	24±2
Na	30000 (n=3)
K	4000
H	0.003 (est Watzke et al., 2000)
***SLC1A2* (EAAT2)**	[1]
Glutamate	97±4
Aspartate	7±1
Na	30000 (n=3)
K	4000 (est equal SLC1A1)
H	0.003 (est)
***SLC1A3* (EAAT1)**	[1]
Glutamate	48±10
Aspartate	16±1
Na	41000 (n=3)
K	4000 (est equal SLC1A1)
H	0.03 (est)
***SLC1A4* (ASCT1)**	[5]
Proline	672±185
Alanine	71±14
Serine	88±11
Cysteine	29±6
Threonine	137±19
Valine	390±8
Na	16000
***SLC1A5* (ASCT2)**	[6]
Asymmetry of K_M	K_{Mi}/K_{Mo}=100 [7]
Alanine	18.4
Serine	18.6
Threonine	20.5
Cysteine	18.8
Glutamine	23.8
Methionine	288

Glycine	361
Leucine	367
Valine	522
Glutamate	1630
Na	5700
***SLC1A6* (EAAT4)**	[8]
Glutamate	2.5±0.9
Aspartate	1±0.3
Na	30000 (est equal SLC1A1)
K	4000 (est equal SLC1A1)
H	0.03 (est)
***SLC1A7* (EAAT5)**	[9]
Glutamate	64±6
Aspartate	13±5
Na	30000 (est equal SLC1A1)
K	4000 (est equal SLC1A1)
H	0.03 (est)

**SLC6 family**	
***SLC6A5* GlyT2**	[10, 11]
Glycine	17
Na	82000 (n=3)
Cl	48500
***SLC6A6* (TAUT)**	[12]
Taurine	5
Na	80000 (n=2)
Cl	30000
***SLC6A7* (PROT)**	[13]
Proline	6.2
Na	80000 (n=2) (est equal TauT)
Cl	30000 (est equal TauT)
***SLC6A9* (GlyT1)**	[11]
Glycine	220
Na	90000 (n=2)
Cl	16000
***SLC6A14* (ATB$^{0,+}$)**	
Amino acid EC_{50}	[14]
Isoleucine	6 ± 1
Leucine	12 ± 2
Methionine	14 ± 1
Valine	36 ± 2
Alanine	99 ± 36
Glycine	111 ± 30
Proline	>5000
Serine	43 ± 5
Cysteine	118 ± 33
Asparagine	348 ± 84
Threonine	405 ± 80
Glutamine	633 ± 62
Phenylalanine	17 ± 1
Tryptophan	26 ± 6
Tyrosine	92 ± 10
Histidine	76 ± 20
Lysine	100 ± 1
Arginine	104 ± 35
Na	7400 (n=2.3)
Cl	920

SLC6A15 **(B⁰AT2)**	[15, 16]
Proline	195±16
Alanine	670±92
Valine	70 (est average Leu/Ile)
Methionine	40±4
Leucine	81±9
Isoleucine	58±10
Phenylalanine	1050±112
Na	16000
SLC6A17 **(NTT4)**	[17]
Leucine	280
Methionine	280 (est equal Leu)
Proline	360
Cysteine	360 (est based on Fig. 5)
Alanine	360 (est based on Fig. 5)
Glutamine	1600
Serine	1600 (est based on Fig. 5)
Histidine	2000 (est based on Fig. 5)
Glycine	2000 (est based on Fig. 5)
Na	23000
SLC6A19 **(B⁰AT1)**	[18, 19]
Methionine	1000 (est based on Fig. 2 Cheng et al.)
Leucine	1100
Isoleucine	1100 (est based on Fig. 2 Cheng et al.)
Valine	4000 (est based on Fig. 2 Cheng et al.)
Glutamine	4000
Asparagine	4000 (est based on Fig. 2 Cheng et al.)
Cysteine	4000 (est based on Fig. 2 Cheng et al.)
Phenylalanine	4700
Alanine	4100
Serine	4000 (est based on Fig. 2 Cheng et al.)
Glycine	11700
Tyrosine	4700 (est equal Phe)
Threonine	4000 (est based on Fig. 2 Cheng et al.)
Histidine	4000 (est based on Fig. 2 Cheng et al.)
Proline	11000 (est equal Gly)
Tryptophan	4700 (est equal Phe)
Na	21000
SLC6A20 **(SIT)**	[20, 21]
Proline	130
Na	22000
Cl	2300

**SLC7 family**	
SLC7A1	[22]
Arginine	77
Lysine	73
Ornithine	105
SLC7A2	[23]
Arginine	2700 (CAT2A) 38 (CAT2B)
Lysine	51
Ornithine	174
SLC7A3	[24]
Arginine	103
Lysine	147
Ornithine	219
SLC7A4	Lysosomal

***SLC7A5* (LAT1)**	[25]
Asymmetry of K_M	K_{Mi}/K_{Mo} = 1000 [26]
Leucine	20
Isoleucine	25
Valine	47
Methionine	20
Phenylalanine	14
Tyrosine	28
Tryptophan	21
Histidine	12
Glutamine	1640
Asparagine	2150
***SLC7A6* (y+LAT2)**	[27, 28]
Arg	138
Lys	138 (est based on Fig. 3 and 5 Broer et al.)
His	138 (est based on Fig. 3 and 5 Broer et al.)
Leu	236
Tyr	500 (est based on Fig. 3 and 5 Broer et al.)
Trp	236 (est based on Fig. 3 and 5 Broer et al.)
Phe	236 (est based on Fig. 3 and 5 Broer et al.)
Gln	295
Met	236 (est based on Fig. 3 and 5 Broer et al.)
Cys	500 (est based on Fig. 3 and 5 Broer et al.)
Ile	500 (est based on Fig. 3 and 5 Broer et al.)
Ala	1000 (est based on Fig. 3 and 5 Broer et al.)
Na	5000
***SLC7A7* (y+LAT1)**	[29, 30]
Arg	340
Lys	68
His	44 (est equal Leu)
Leu	44
Tyr	200 (est based on Fig. 8 Kanai et al.)
Phe	300 (est based on Fig. 8 Kanai et al.)
Gln	60 (est based on Fig. 3 Pfeiffer et al.)
Met	60 (est based on Fig. 3 Pfeiffer et al.)
Ile	60 (est based on Fig. 8 Kanai et al.)
Na	15000 (est)
***SLC7A8* (LAT2)**	[31]
Asymmetry of K_M	K_{Mi}/K_{Mo} = 200 [26]
Histidine	181
Tryptophan	58
Tyrosine	36
Phenylalanine	45
Valine	124
Isoleucine	97
Leucine	119
Methionine	204
Glutamine	151
Asparagine	81
Cysteine	109
Threonine	69
Serine	116
Alanine	187
Glycine	265 (Low Vmax 21%)
***SLC7A9* ($b^{0,+}$AT)**	[32]
Asymmetry of K_M	K_{Mi}/K_{Mo} = 20 [33]
Arginine	108

Lysine	394
Ornithine	195
Cystine	296
Alanine	300 (est based on Fig. 6 Mizoguchi et al. 2001)
Cysteine	100 (est based on Fig. 6 Mizoguchi et al. 2001)
Phenylalanine	100 (est based on Fig. 6 Mizoguchi et al. 2001)
Histidine	500 (est based on Fig. 6 Mizoguchi et al. 2001)
Isoleucine	400 (est based on Fig. 6 Mizoguchi et al. 2001)
Leucine	107
Valine	100 (est based on Fig. 6 Mizoguchi et al. 2001)
Methionine	100 (est based on Fig. 6 Mizoguchi et al. 2001)
Asparagine	500 (est based on Fig. 6 Mizoguchi et al. 2001)
Glutamine	500 (est based on Fig. 6 Mizoguchi et al. 2001)
Serine	150 (est based on Fig. 6 Mizoguchi et al. 2001)
Threonine	100 (est based on Fig. 6 Mizoguchi et al. 2001)
Tryptophan	500 (est based on Fig. 6 Mizoguchi et al. 2001)
Tyrosine	150
***SLC7A10* (asc-1)**	[34]
Alanine	23
Glycine	8
Serine	11
Threonine	19
Cysteine	24
Valine	112
Methionine	139
Isoleucine	160
Leucine	245
Histidine	368
Phenylalanine	464
***SLC7A11* (xCT)**	[35]
Glutamate	92
Cystine	43
***SLC7A12* (AGT-1)**	[36, 37]
Aspartate	25
Glutamate	21
Cystine	68

**SLC16 family**	
SLC16A10	[38]
Tryptophan	3720
Tyrosine	2590
Phenylalanine	7020

**SLC36 family**	
***SLC36A1* (PAT1)**	[39]
Glycine	7000
Proline	2800
Alanine	7500
H	0.3
***SLC36A2* (PAT2)**	[39]
Glycine	590
Proline	120
Alanine	260
H	0.01
SLC36A3	Not characterised
SLC36A4	[40]
Proline	3

Alanine	1480
Tryptophan	2
H	0.3 (est equal PAT1)

**SLC38 family**	
***SLC38A1* (SNAT1)**	[41, 42]
Ala	306
Ser	306 (est based on Fig. 2 Albers et al.)
Thr	890 (est based on Fig. 2 Albers et al.)
Cys	306 (est based on Fig. 2 Albers et al.)
Met	200 (est based on Fig. 2 Albers et al.)
His	230 (est based on Fig. 2 Albers et al.)
Gln	230
Asn	306 (est based on Fig. 2 Albers et al.)
Gly	890 (est based on Fig. 2 Albers et al.)
Pro	1000 (est based on Fig. 2 Albers et al.)
Na	9500
***SLC38A2* (SNAT2)**	[42, 43]
Ala	529
Ser	529 (est based on Fig. 2 MacKenzie et al. 2003)
Thr	1900 (est based on Fig. 2 MacKenzie et al. 2003)
Cys	529 (est based on Fig. 2 MacKenzie et al. 2003)
Met	800 (est based on Fig. 2 MacKenzie et al. 2003)
His	800 (est based on Fig. 2 MacKenzie et al. 2003)
Gln	1650
Asn	600 (est based on Fig. 2 MacKenzie et al. 2003)
Gly	800 (est based on Fig. 2 MacKenzie et al. 2003)
Pro	800 (est based on Fig. 2 MacKenzie et al. 2003)
Na	20000
***SLC38A3* (SNAT3)**	[44, 45]
Glutamine	1500
Histidine	7400
Asparagine	1300
Na	15000
H	0.1
***SLC38A4* (SNAT4)**	[46, 47]
Ala	4200
Arg	300 ± 40
Ser	4000 (est based on Fig. 3 Sugawara et al., 2000)
Gly	1600 ± 300
Cys	4000 (est based on Fig. 3 Sugawara et al., 2000)
Asn	3000 (est based on Fig. 3 Sugawara et al., 2000)
Thr	3000 (est based on Fig. 3 Sugawara et al., 2000)
Pro	3000 (est based on Fig. 3 Sugawara et al., 2000)
Met	3000 (est based on Fig. 3 Sugawara et al., 2000)
Gln	2500 ± 500
Na	70000
***SLC38A5* (SNAT5)**	[48]
Glycine	15200
Asn	2500
Ala	2500 (est equal Asn)
Ser	2500 (est equal Asn)
Gln	3200
His	600
Na	11000
H	0.03
SLC38A6	Uncharacterised

SLC38A7	Lysosomal
SLC38A8	Uncharacterised
SLC38A9	Lysosomal
SLC38A10	ER/Golgi

**SLC43 family**	
SLC43A1 **(LAT3)**	[49]
Leucine	1024 ± 31
Isoleucine	1418 ± 47
Valine	1885 ± 107
Phenylalanine	1206 ± 53
Methionine	2000 est
SLC43A2 **(LAT4)**	[50]
Leucine	3733
Isoleucine	4000 (est between Leu and Phe)
Valine	4000 (est between Leu and Phe)
Phenylalanine	4694
Methionine	4000 (est between Leu and Phe)

List S4: Equations for different types of amino acid transporters

Preparatory calculations for all transporters:

Fractional saturation:

Exofacial (ext=extracellular):

$$F_{iext} = \left(\frac{AAi_{ext}}{Km_{appext} + AAi_{ext}}\right)$$

Endofacial (cyt=cytosolic):

$$F_{icyt} = \left(\frac{AAi_{cyt}}{Km_{appcyt} + AAi_{cyt}}\right)$$

Total saturation $F_T = \sum F_i$

$F_{AAi}=F_i/F_T$

1) **Electroneutral Uniporter: SLC16A10, SLC43A1, SLC43A2**

J_{Tec}=Total flux from extracellular (e) medium to cytosol (c)

J_{Tce}=Total flux from cytosol (c) to extracellular medium (e)

$$J_{Tec} = J_{max}(F_{Text})$$

$$J_{Tce} = J_{max}(F_{Tcyt})$$

2) **Electrogenic Uniporter (cationic AA): *SLC7A1*, *SLC7A2*, *SLC7A3***

$$J_{Tec} = J_{max}(F_{Text})\left(\beta(1 - F_{Tcyt})\right)$$

$$J_{Tce} = J_{max}(F_{Tcyt})\left(\frac{1}{\beta}(1 - F_{Text})\right)$$

3) **Na^+-Symporter: SLC6A15, SLC6A19, SLC38A1, SLC38A2, SLC38A4**

$$J_{Tec} = J_{max}\left((F_{Text}) \left(\frac{Na_{ext}}{Km_{ext} + Na_{ext}}\right) \left(\beta \left(1 - F_{Tcyt}\right) \left(\frac{Km_{cyt}}{Km_{cyt} + Na_{cyt}}\right)\right)\right)$$

$$J_{Tce} = J_{max}\left(\left(F_{Tcyt}\right) \left(\frac{Na_{cyt}}{Km_{cyt} + Na_{cyt}}\right) \left(\frac{1}{\beta}\right) (1 - F_{Text}) \left(\frac{Km_{ext}}{Km_{ext} + Na_{ext}}\right)\right)$$

4) Complex symporter 1: SLC1A1, SLC1A2, SLC1A3, SLC1A6, SLC1A7

$$J_{Tec} = J_{max}\, (F_{Text}) \left(\frac{(Na_{ext})^3}{(Km_{ext})^3 + (Na_{ext})^3}\right) \left(\frac{(H^+{}_{ext})}{Km_{ext} + (H_{ext})}\right) \left(\frac{\left(K_{cyt}\right)}{Km_{cyt} + \left(K_{cyt}\right)}\right) * \beta\left(1 - F_{Tcyt}\right) \left(\frac{(Km_{cyt})^3}{(Km_{cyt})^3 + (Na_{cyt})^3}\right) \left(\frac{Km_{cyt}}{Km_{cyt} + H_{cyt}}\right) \left(\frac{Km_{ext}}{Km_{ext} + K_{ext}}\right)$$

$$J_{Tce} = J_{max}\left(F_{Tcyt}\right) \left(\frac{(Na_{cyt})^3}{(Km_{cyt})^3 + (Na_{cyt})^3}\right) \left(\frac{(H^+{}_{cyt})}{Km_{cyt} + (H_{cyt})}\right) \left(\frac{(K_{ext})}{Km_{ext} + (K_{ext})}\right) * \frac{1}{\beta} (1 - F_{Text}) \left(\frac{(Km_{ext})^3}{(Km_{ext})^3 + (Na_{ext})^3}\right) \left(\frac{Km_{ext}}{Km_{ext} + H_{ext}}\right) \left(\frac{Km_{cyt}}{Km_{cyt} + K_{cyt}}\right)$$

5) Complex symporter 2: SLC6A7, SLC6A9, SLC6A14, SLC6A17, SLC6A18, SLC6A20

$$J_{Tec} = J_{max}\, (F_{Text}) \left(\frac{(Na_{ext})^2}{(Km_{ext})^2 + (Na_{ext})^2}\right) \left(\frac{(Cl^-{}_{ext})}{Km_{ext} + Cl^-_{ext})}\right) * \beta\left(1 - F_{Tcyt}\right) \left(\frac{(Km_{cyt})^2}{(Km_{cyt})^2 + (Na_{cyt})^2}\right) \left(\frac{Km_{cyt}}{Km_{cyt} + Cl_{cyt}}\right)$$

$$J_{Tce} = J_{max}\left(F_{Tcyt}\right) \left(\frac{(Na_{cyt})^2}{(Km_{cyt})^2 + (Na_{cyt})^2}\right) \left(\frac{(Cl_{cyt})}{Km_{cyt} + (Cl_{cyt})}\right) * \frac{1}{\beta} (1 - F_{Text}) \left(\frac{(Km_{ext})^2}{(Km_{ext})^2 + (Na_{ext})^2}\right) \left(\frac{Km_{ext}}{Km_{ext} + Cl_{cyt}}\right)$$

6) Complex symporter 3: SLC6A5

$$J_{Tec} = J_{max}\left(F_{Tcyt}\right) \left(\frac{(Na_{ext})^3}{(Km_{ext})^3 + (Na_{ext})^3}\right) \left(\frac{(Cl^-{}_{ext})}{Km_{appext} + Cl^-_{ext})}\right) * \beta\left(1 - F_{Tcyt}\right) \left(\frac{(Km_{cyt})^3}{(Km_{cyt})^3 + \left(Na_{cyt}\right)^3}\right) \left(\frac{Km_{cyt}}{Km_{cyt} + Cl_{cyt}}\right)\Bigg)$$

$$J_{Tce} = J_{max}\left(F_{Tcyt}\right) \left(\frac{(Na_{cyt})^3}{(Km_{cyt})^3 + (Na_{cyt})^3}\right) \left(\frac{(Cl_{cyt})}{Km_{cyt} + (Cl_{cyt})}\right) * \frac{1}{\beta} (1 - F_{Text}) \left(\frac{(Km_{ext})^3}{(Km_{ext})^3 + (Na_{ext})^3}\right) \left(\frac{Km_{ext}}{Km_{ext} + Cl_{cyt}}\right)$$

7) Electroneutral symporter: SLC38A3, SLC38A5

$$J_{Tec} = J_{max}\, (F_{Text}) \left(\frac{(Na_{ext})}{Km_{ext} + Na_{ext}}\right) \left(\frac{\left(H^+{}_{cyt}\right)}{Km_{cyt} + (H^+_{cyt})}\right)$$

$$J_{Tce} = J_{max}\left(F_{Tcyt}\right) \left(\frac{\left(Na_{cyt}\right)}{Km_{cyt} + (Na_{cyt})}\right) \left(\frac{(H^+{}_{ext})}{Km_{ext} + (H^+_{ext})}\right)$$

8) Proton-amino acid symporter: SLC36A1, SLC36A2, SLC36A3, SLC36A4

$$J_{Tec} = J_{max}\, (F_{Text}) \left(\frac{H_{ext}}{Km_{ext} + H_{ext}}\right) \beta\left(1 - F_{Tcyt}\right) \left(\frac{Km_{cyt}}{Km_{cyt} + H_{cyt}}\right)$$

$$J_{Tce} = J_{max}\left(F_{Tcyt}\right) \left(\frac{H_{cyt}}{Km_{cyt} + H_{cyt}}\right) \left(\frac{1}{\beta}\right) (1 - F_{Text}) \left(\frac{Km_{ext}}{Km_{ext} + H_{ext}}\right)$$

9) Antiporter: SLC1A4, SLC1A5, SLC7A5, SLC7A8, SLC7A10, SLC7A11, SLC7A12

$$J_{Tec} = J_{max}(F_{Text})(F_{Tcyt})$$

$$J_{Tce} = J_{Tec}$$

10) Complex antiporter: SLC7A6, SLC7A7

$$J_{Tec} = J_{max}(F_{Text})(F_{Tcyt})$$

$$J_{Tce} = J_{max}(F_{Text})(F_{Tcyt})$$

If AAi is cationic: $Fractional\ saturation\ (F_{iext}) = \left(\frac{AAi_{ext}}{Km_{appext}+AAi_{ext}}\right)$ and $F_{icyt} = \left(\frac{AAi_{cyt}}{Km_{appcyt}+AAi_{cyt}}\right)$

IfAAi is neutral: $Fractional\ saturation\ (F_{iext}) = \left(\frac{AAi_{ext}}{Km_{appext}+AAi_{ext}}\right)\left(\frac{Na_{ext}}{Km_{ext}+Na_{ext}}\right)$ and $F_{icyt} = \left(\frac{AAi_{cyt}}{Km_{appcyt}+AAi_{cyt}}\right)\left(\frac{Na_{cyt}}{Km_{cyt}+Na_{cyt}}\right)$

11) Electrogenic antiporter: SLC7A9

	Substrate extracellular	Substrate cytosolic
Mode 1	Cationic	Cationic
Mode 2	Cationic	Neutral
Mode 3	Neutral	Cationic
Mode 4	Neutral	Neutral

Equation for Mode 1 and 4: $J_{iec} = J_{max}(F_{i1ext})(F_{i2cyt})$

Equation for Mode2: $J_{iec} = J_{max}(F_{i1ext})(F_{i2cyt})\beta$

Equation for Mode 3: $J_{iec} = J_{max}(F_{i1ext})(F_{i2cyt})(\frac{1}{\beta})$

In each case: $J_{aa1ce} = J_{aa2ec}$

Table S5: Predicted and simulated maximal accumulation of substrates by different types of amino acid transporters.

Type of transporter	Theoretical accumulation	Simulated accumulation
Uniporter	1	1
Uniporter electrogenic	10.34	10.34
1Na+ AA Cotransporter	103.44	103.44
3Na+/1H+ AA Cotransporter/ K+ antiporter	2568342	1712230*
2Na+/1Cl- AA Cotransporter	1410.65	1410.65
3Na+/1Cl- AA Cotransporter	145928.5	145928.5
1Na+ AA Cotransporter/H+ antiporter	6.31	5.49*
1H+ AA cotransporter	8.22	8.22

* Where antiport processes are involved the simulated accumulation is less than the maximal theoretical accumulation. There are no experimental data available to support or refute this result.

Table S6: Calculated Vmax values for cell lines used in this study

Vmax values are given as nmol/min*mg protein and were calculated *Vmax=v*(Km+[S])/([S]*min)*.

Green fields were used to calculate averages. The table also includes metabolic conversions used in the simulation as %/iteration. For myotubes mRNA expression levels were used instead of calculated Vmax.

Transporter	Probe AA			A549		
SLC1		Conc	min	Activity	Km	Vmax
EAAT3	Glu	100	6		62	
EAAT1	Glu	100	6		48	
EAAT2	Glu	100	6		48	
EAAT4	Glu	100	6		3	
EAAT5	Glu	100	6		64	
ASCT1	Ala	300	2		71	
	Pro	100	2	1.2	672	4.632
ASCT2	Ala	300	2	48	18	25.44
	Gln	100	2	23	24	14.26
	Gly	100	6	1.5	361	1.1525
SLC6						
GlyT2	Gly	100	6		17	
GlyT1	Gly	100	6		220	
PROT	Pro	100	6		6	
ATB0,+	Leu	100	2		12	
	Arg	100	6		104	
B0AT2	Leu	100	2		81	
	Ala	300	2		670	
	Pro	100	6		195	
NTT4	Gln	100	2		5200	
	Leu	100	2		280	
	Pro	100	6		360	
B0AT1	Leu	100	2		1000	
	Ala	300	2		4100	
	Gln	100	2		3200	
SIT1	Pro	100	6		130	
SLC7						
CAT1	Arg	100	6	10	77	2.95
CAT2	Arg	100	6		2700	
CAT3	Arg	100	6		103	
LAT1	Leu	100	2	26	20	15.6
	Gln	100	2	3.2	1640	27.84
y+LAT2	Leu	100	2	6.4	236	10.752
	Arg	100	6	15	138	5.95
y+LAT1	Leu	100	2		44	
	Arg	100	6		340	
LAT2	Leu	100	2	12	119	13.14
	Ala	300	2	11.3	187	9.171833
	Gln	100	2	6.8	151	8.534
b0,+AT	Leu	100	2		107	
	Arg	100	6		108	
Asc-1	Ala	300	2		23	
xCT	Glu	25	6	4.8	92	3.744
AGT1	Glu	100	6		21	
SLC16						
TAT1	Phe	100			7020	
SLC36						
PAT1	Gly	100	6		7000	
	Pro	100	6		2800	
PAT2	Gly	100	6		590	
	Pro	100	6		120	
PAT4	Pro	100	6		3	
	Ala	300	2		1480	
SLC38						
SNAT1	Ala	300	2	9	306	9.09
	Gln	100	2	4.5	230	7.425
	Pro	100	6	7.8	1000	14.3
	Gly	100	6	2.1	890	3.465
SNAT2	Ala	300	2	1.3	529	1.796167
	Gln	100	2	0.6	1650	5.25
	Leu	100	2	5.3	1000	29.15
	Pro	100	6	3.6	800	5.4
	Gly	100	6	0.2	800	0.3
SNAT3	Gln	100	2		1500	
SNAT4	Ala	300	2		4200	
	Pro	100	6			
	Gln	100	2		2500	
	Arg	100	6	3.6	300	2.4
SNAT5	Ala	300	2	6.9	2500	32.2
	Gln	100	2	4.3	3200	70.95
	Gly	100	6		15200	
SNAT7	Gln	100	2			
SNAT8	Gln	100	2			
SLC43						
LAT3	Leu	100	2		1024	
	Phe	100	2		1206	
LAT4	Leu	100	2		3733	
	Phe	100	2		4694	
Amino acid conversion:		Gln/Glu	0.001			
		Cysn/Cys	1			

Transporter	Probe AA			U87-MG		
SLC1		Conc	min	Activity	Km	Vmax
EAAT3	Glu	100	6	0.37	62	0.0999
EAAT1	Glu	100	6		48	
EAAT2	Glu	100	6		48	
EAAT4	Glu	100	6		3	
EAAT5	Glu	100	6		64	
ASCT1	Ala	300	2	2.7	71	1.6695
	Pro	100	2		672	
ASCT2	Ala	300	2	14.7	18	7.791
	Gln	100	2	6	24	3.72
					361	
SLC6						
GlyT2	Gly	100	6		17	
GlyT1	Gly	100	6		220	
PROT	Pro	100	2		6	
ATB0,+	Leu	100	2		12	
	Arg	100	6		104	
B0AT2	Leu	100	2	1.64	81	1.4842
	Ala	300	2	6.54	670	10.573
	Pro	100	2	1.48	195	2.183
NTT4	Gln	100	2		5200	
	Leu	100	2		280	
	Pro	100	2		360	
B0AT1	Leu	100	2		1000	
	Ala	300	2		4100	
	Gln	100	2		3200	
SIT1	Pro	100	2		130	
SLC7						
CAT1	Arg	100	6	1.56	77	0.4602
CAT2	Arg	100	6		2700	
CAT3	Arg	100	6		103	
LAT1	Leu	100	2	18.4	20	11.04
	Gln	100	2	1.03	1640	8.961
y+LAT2	Leu	100	2	2.29	236	3.8472
	Arg	100	6	5.92	138	3.3152
y+LAT1	Leu	100	2		44	
	Arg	100	6		340	
LAT2	Leu	100	2	0	119	0
	Ala	300	2	0.8	187	0.649333
	Gln	100	2	0.45	151	0.56475
b0,+AT	Leu	100	2		107	
	Arg	100	6		108	
Asc-1	Ala	300	2		23	
xCT	Glu	100	6	0.7	92	0.224
AGT1	Glu	100	6		21	
SLC16						
TAT1	Phe	100			7020	
SLC36						
PAT1	Gly	100	6		7000	
	Pro	100	6		2800	
PAT2	Gly	100	6		590	
	Pro	100	6		120	
PAT4	Pro	100	6		3	
	Ala	300	2		1480	
SLC38						
SNAT1	Ala	300	2	7.59	306	7.6659
	Gln	100	2	1.69	230	2.7885
	Pro	100	6		1000	
	Gly	100	6		890	
SNAT2	Ala	300	2	3	529	4.145
	Gln	100	2	0	1650	0
	Leu	100	2	0.45	1000	2.475
	Pro	100	6		800	
	Gly	100	6		800	
SNAT3	Gln	100	2		1500	
SNAT4	Ala	300	2	3.62	4200	27.15
	Pro	100	6	0.53	4200	3.798333
	Gln	100	2	5.05	2500	65.65
	Arg	100	6	1.24	300	0.826667
SNAT5	Ala	300	2	3.9	2500	18.2
	Gln	100	2	2.9	3200	47.85
	Gly	100	2		15200	
SNAT7	Gln	100	2			
SNAT8	Gln	100	2			
SLC43						
LAT3	Leu	100	2		1024	
	Phe				1206	
LAT4	Leu	100	2		3733	
	Phe				4694	
Amino acid conversion:		Gln/Glu	0.0003			
		Cysn/Cys	1			

	**Myotubes**
**Transporter**	**Vmax**
SLC1	
EAAT3	0.0046
EAAT1	0.1353
EAAT2	0.0012
ASCT1	0.2633
ASCT2	0.621
EAAT4	0
EAAT5	0
SLC6	
GlyT2	0
GlyT1	0.1399
PROT	0
ATB0,+	0
B0AT2	0.0233
NTT4	0.0602
XT2	0
B0AT1	0
SIT1	0
SLC7	
CAT1	0.2181
CAT2	0.3201
CAT3	0
CAT4	0.0133
LAT1	0.7842
y+LAT2	0.1504
y+LAT1	0.3596
LAT2	0.0066
b0,+AT	0
Asc-1	0
xCT	0.1178
Asc-2	0
AGT1	0
SLC16	
TAT1	0.0009
SLC36	
PAT1	0.1118
PAT2	0
PAT4	0.134
SLC38	
SNAT1	0.4219
SNAT2	1.4933
SNAT3	0.0483
SNAT4	0.0195
SNAT5	0.187
SNAT7	0.0773
SNAT8	0
SLC43	
LAT3	0.041
LAT4	0.2367

Table S7 Chemicals, Media, recombinant proteins and assay kits.

Product	Source	Identifier
DMEM/F-12, no glutamine	Thermo Fisher Scientific	21331-020
DMEM, high glucose, pyruvate	Thermo Fisher Scientific	11995
DMEM w/o glucose, amino acids, sodium bicarbonate, sodium pyruvate, phenol red (powder)	US Biological	D9800-20A
Basal Medium Eagle (BME)	Thermo Fisher Scientific	21010-046
Skeletal Muscle Cell Growth Basal Medium-2	Lonza	CC-3246
Fetal Bovine Serum, Performance Plus (US origin)	Thermo Fisher Scientific	16000-044
Fetal Bovine Serum, dialyzed (US origin)	Thermo Fisher Scientific	26400-044
Horse Serum, heat inactivated (New Zealand origin)	Thermo Fisher Scientific	26050-088
SKGM-2 SingleQuots Supplements and Growth Factors	Lonza	CC-3244
L-Glutamine (200mM)	Thermo Fisher Scientific	25030-081
Sodium Pyruvate (100mM)	Thermo Fisher Scientific	11360-070
Trypsin-EDTA (0.25%), phenol red	Thermo Fisher Scientific	25200-072
Trypsin-EDTA (0.05%), phenol red	Thermo Fisher Scientific	25300-062
ReagentPack Subculture Reagents	Lonza	CC-5034
Penicillin-Streptomycin (10 000 U/mL P, 10mg/ml S)	Thermo Fisher Scientific	15140-122
Dimethyl sulfoxide	Sigma-Aldrich	D8418
Corning BioCoat Collagen I-coated Microplates	Corning	152036
Palmitic Acid	MP Biomedicals	100905
Oleic Acid	Sigma Aldrich	O1383
D-glucose	Sigma-Aldrich Inc.	G8644
TNF-α	Thermo Fisher Scientific	PHC3015
RIPA buffer	Cell Signaling Technology	9806
EZ-Link Sulfo-NHS-LC-Biotin	Thermo Fisher Scientific	21335
cOmplete, mini, EDTA-free protease inhibitor cocktail	Roche Diagnostics GmbH	11836170001
High capacity streptavidin agarose resin	Thermo Fisher Scientific	20359
SeeBlue Plus2 prestained standard	Thermo Fisher Scientific	LC5925
Pierce ECL Western Blotting Substrate	Thermo Fisher Scientific	32209
SuperSignal West Femto Trial Kit	Thermo Fisher Scientific	34094
[^{14}C(U)]-Glycine, >97%, 250µCi	PerkinElmer	NEC276E250UC
[^{14}C(U)]-Alanine, >97%, 50µCi	PerkinElmer	NEC266E050UC
[^{14}C(U)]-Leucine, >97%, 250µCi	PerkinElmer	NEC279E250UC
[^{14}C(U)]-Proline, >97%, 50µCi	PerkinElmer	NEC285E050UC
[^{14}C(U)]-Glutamine, >97%, 50µCi	PerkinElmer	NEC451050UC
[^{14}C(U)]-Glutamic acid, >97%, 50µCi	PerkinElmer	NEC290E050UC
[^{14}C(U)]-Arginine, >97%, 50µCi	PerkinElmer	NEC267E050UC
α-(methylamino)isobutyric acid (MeAIB)	Sigma Aldrich	M2383
JPH203 Dihydrochloride	MedChemExpress	HY-U00445
CB-839 (Telaglenastat)	MedChemExpress	HY-12248
2-amino-2-norbornanecarboxylic acid (BCH)	Sigma Aldrich	A7902
Loratadine	Sigma Aldrich	L9664
UCPH-101	Cayman Chemical Company	21460
Erastin	Sigma Aldrich	E7781
DL-threo-β-hydroxyaspartic acid	Sigma Aldrich	H2775
Org 25543 hydrochloride	Tocris Bioscience	4782
Sulfasalazine	Cayman Chemical Company	15025
Dihydrokainic acid	Abcam	ab120066
N-ethylmaleimide	Sigma Aldrich	E3876
Ultima Gold	PerkinElmer	6013329
Amino Acid Standard	Sigma Aldrich	AAS18
Canonical amino acid mix	Cambridge Isotope Laboratories	MSK-CAA-1

Metabolomics amino acid mix standard	Cambridge Isotope Laboratories	MSK-A2-1.2
L-glutamine (^{13}C-5, 99%)	Cambridge Isotope Laboratories	CLM-1822-H-0.1MG
Adonitol (ribitol)	Sigma Aldrich	A5502
Pyridine	Sigma Aldrich	270970
Methoxyamine hydrochloride	Supelco	33045-U
N-(*tert*-butyldimethylsilyl)-*N*-methyltrifluoroacetamide with 1% *tert*-butyldimethylchlorosilane	Sigma Aldrich	00942
Alkanes	Sigma Aldrich	68281
Bradford reagent	Sigma Aldrich	B6916
SuperScript II reverse transcriptase	Invitrogen	18064014
100 bp DNA ladder	New England BioLabs	N3231S
RNeasy Mini Kit (50)	Qiagen	74104
Taq DNA Polymerase (1000 U)	Qiagen	201205
BCA assay	Sigma	B9643

Table S8 Cell lines and primary cells.

**Experimental Models: Cell Lines**	**Source**	**Identifier**
A549	Gifted by Ross Hannan	N/A
U87MG (from Glioma Tumor Cell Panel)	ATCC	TCP-1018
Muscle Myoblasts SKGM-2	Lonza	CC-2580

Table S9 Software and scripts

**Software and scripts**	**Source**	**Identifier**
Origin 2018b	OriginLab	N/A
Microsoft Office Professional Plus 2019	Microsoft Corporation	N/A
MatLab 2019b	Mathworks	N/A
Thermo Xcalibur 4.1	Thermo Fisher Scientific	N/A
TraceFinder 3.3	Thermo Fisher Scientific	N/A
DExSI 1.11	https://github.com/DExSI/DExSI/	N/A
Image J	https://imagej.nih.gov/ij/	N/A
Oncomine	https://www.oncomine.org	N/A
JDFC_v1.1	stefanbroeer/JDFC_v1.1: Updated version of JDFC (github.com)	JDFC
Prism 9.0	Graphpad	N/A
Biorender	Biorender.com	Images 1, 6h, 8a were created using Biorender

Table S10 Amino acid concentrations used for PHM treatment. PHM media baseline AA concentrations (0.5x). Concentrations match mean physiological levels found in human plasma (values published in the Human Metabolome Database, HMDB; LIVRQNacHKFT (bold) were added at specified fold concentrations above plasma level (2x/5x; Nac is not endogenous in plasma and was proportionally scaled between 0.2/0.5 mM)

**Amino Acid**	**[0.5x] (mM)**	**[2x] (mM)**	**[5x] (mM)**
Glycine	0.126	0.126	0.126
Alanine	0.224	0.224	0.224
**Arginine**	0.054	0.272	0.598
Asparagine	0.024	0.024	0.024
Aspartate	0.01	0.01	0.01
Cysteine	0.037	0.037	0.037
Glutamate	0.032	0.032	0.032

**Glutamine**	0.281	1.405	3.092
Histidine	0.05	0.251	0.553
**Isoleucine**	0.033	0.166	0.365
**Leucine**	0.076	0.381	0.84
Lysine	0.109	0.546	1.202
Methionine	0.014	0.014	0.014
Phenylalanine	0.037	0.185	0.408
Proline	0.095	0.095	0.095
Serine	0.071	0.071	0.071
Threonine	0.075	0.377	0.83
Tryptophan	0.03	0.03	0.03
Tyrosine	0.038	0.038	0.038
**Valine**	0.117	0.585	1.288
**N-acetylcysteine**	**N/A**	0.2	0.5

Table S11 RT-PCR primers used in this study.

**Transporter**	**Forward Primer**	**Reverse Primer**	**Fragment size bp**
**SLC1**			
EAAT3 (A1)	CCTGGTGTCACCCAGAAAGT	TTGCGGAATATTTCCCAGTC	491
EAAT2 (A2)	ATCAAGAAGGGCCTGGAGTT	TCCAACAGGAAGGACGAATC	492
EAAT1 (A3)	CATGCACAGAGAAGGCAAAA	GTCACGGTGTACATGGCAAG	498
ASCT1 (A4)	GATCCCCATAGGCACTGAGA	TGGGGAGAATAAACCTGCTG	485
ASCT2 (A5)	GCAGCCTTTCGCTCATACTC	GGGCAAAGAGTAAACCCACA	307
EAAT4 (A6)	CACCTACCGCCAGATCAAGT	GCTCAGACCCGTTCTCTGTC	416
EAAT5 (A7)	TCCTCATCCTGTCTGTGCTG	CTTGACAACTGGGGTGGTCT	449
SLC3			
rBAT (A1)	CCAGTTGGCACTTTGACGAA	AGTGTCTAGCATGCTGAGGT	470
4F2hc (A2)	GAGCCGAGAAGAATGGTCTG	AGCCGATTGCAAGAGACTGT	481
SLC6			
GlyT1 (A5)	GAGAAACTCACGGATGCCAC	AGCAGCCCAGAGTAAACACT	454
PROT (A7)	CCCAGTTCCACCACTTGTTG	AGATGAGCCCTGAGAACACC	461
GlyT2 (A9)	CAGACTCATTTACGTGGGGG	GGTGCATGGTAATCGGCTTT	443
ATB^{0+} (A14)	AGAGGCTCTAGCCCAACTCC	AGCTCTCCACCATAGCCAGA	394
B^{0}AT2 (A15)	TCTGGGATTTGGTGGTGTCA	AAGGCCTAGATTGACCAGCA	484
NTT5 (A16)	GCCTCTTCTTCACTCGACCT	TGGTGACTTAGGGGTAGGGA	475
NTT4 (A17)	TCAACTTCTCCCACCTGACC	CCACAGCGATGTTCTCAAGG	457
B^{0}AT3 (A18)	GCTCTCTAGTGCTGGGTGTG	TGTTCTGTTGAGGTCCGGTG	504
B^{0}AT1 (A19)	TACGGTGTGGACAGGTTCAA	TACTTCAGGTCCCCGTTCA	377
IMINO (A20)	CCAACTCGCTACAGTTCGTG	AGGGACGGCGAGATATTGAG	454
SLC7			
CAT1 (A1)	CTATGGCGAGTTTGGTGCTC	TCAAACAGAGACGGCCTGAT	452
CAT2 (A2)	CTGCTATGCCGAATTTGGGG	TTCAGAAGGTGGCTCTCTGG	463
CAT3 (A3)	CACTCAACTCCATCCCCACT	AGTGGACATAGAGAGTGCCG	462
CAT4 (A4)	CTGGGCTTCTTGGATGGGTA	ATAGCCTGCACTGTCTCCTC	464
LAT1 (A5)	GAAGGCACCAAACTGGATGT	AGAGCAGGGTCATCACACAC	460
y^{+}LAT2 (A6)	GGCATGTTCAGCTGGACCAT	TAGCAGCCAGGACATTCCGA	520
y^{+}LAT1 (A7)	GATAATTCCACTGTCAGTTG	CCACGATCCTTCGGAGGTAA	500
LAT2 (A8)	AACCTTCCCAGAGCCATCTT	GGGGGATATCAGGCTTCTTC	473
asc (A10)	GACACTTCGAGGAGCTGAGG	GTGTCTGACGTGGATCATGG	449
xCT (A11)	CCATGGCCATTGTCACCATT	GGATGAACAGTGGCACCTTG	465

SLC16			
TAT1 (A10)	GTGTGGGCAGTTGGAATACC	GAGCCCAGTTTGTCACGAAG	458
SLC36			
PAT1 (A1)	GGGAAGACGTGTTGTGGACT	TCCGAGGATCCTTCATTTTG	523
PAT2 (A2)	ACGTCCCTGCAGAAATCATC	ACCGAACAAAAGTGGTGGAG	375
PAT3 (A3)	CCCAGCAACCTACCCTTGAT	GGATGAGGATGGCTGAGACA	445
PAT4 (A4)	CAGCTGGGATTCTGTAGTGTT	CCCATGCCAATATTCAACCGC	449
SLC38			
SNAT1 (A1)	GAACACTGGAGCAATGCTGA	AACAAACATGGCGAAAAAGG	490
SNAT2 (A2)	GTGGCCTTTCCTTGTTGTGT	TGTCAGGGTCACAGCCATTA	494
SNAT3 (A3)	TGCAGCACGAGATGGTGGAG	GTAGCCCAGCTGCTCATAGG	389
SNAT4 (A4)	ATGGAGAAGTTGAAGATGAA	GATCATAAATCCAGTCAATT	450
SNAT5 (A5)	GCCATGTTCTGCATGTATGG	GGGTACAATGCGGAGGTAGA	381
SNAT6 (A6)	AAAGCTGTAACAATGATGTT	ATGAGTGCTAAACTAAAATT	290
SNAT7 (A7)	CTGACCTTTGGAGCTGCTGT	CTGTGGTCTGGCCGAAGATG	460
SNAT8 (A8)	CTACCCAGGCAATGATATGG	ACTAGCTGCCCATCAGAACA	460
SNAT9 (A9)	TTCAGTTTCCACAGCTGACT	ATGAATACAAAGGCCAGTCC	490
SNAT10 (A10)	TTGCTGGACCAGCAGGAGAA	CGCCAGTAACGTCCTGACTT	520
SNAT11 (A11)	ACCCACAGTAGCTAAGTGGT	CTGCTGAACATGAGACTCTG	610
SLC43			
LAT3 (A1)	CCTTCATGTCACCCCAGGAT	GGCATTGGTGAGCTTTTGGA	437
LAT4 (A2)	GTCTCCTTCATCGTCGTCCT	GGTGTAGAGGCCAACTGTCT	480
Control			
Clathrin (clh)	GACAGTGCCATCATGAATCC	TTTGTGCTTCTGGAGGAAAGA	724

Table S12 Primary and secondary antibodies used in this study.

**Primary antibodies**	**Source**	**Identifier**	**Dilution**
EAAT3 (SLC1A1)	Gifted by David Pow	N/A	1:1000
EAAT1 (SLC1A3)	Abcam	ab416	1:1000
ASCT1 (SLC1A4)	Cell Signaling	8442	1:1000
ASCT2 (SLC1A5)	Cell Signaling	D7C12	1:2000
GlyT2 (SLC6A9)	Abcam	ab99098	1:1000
B0AT2 (SLC6A15)	Pineda Antibody Service	Custom antibody	1:4000
CAT1 (SLC7A1)	MyBioSource	MBS176763	0.5ug/mL
LAT1 (SLC7A5)	Cell Signaling	5347	1:2000
y+LAT2 (SLC7A6)	Abcam	ab235054	1:1000
LAT2 (SLC7A8)	Abcam	ab123896	1:1000
xCT (SLC7A11)	Abcam	ab175186	1:2000
SNAT1 (SLC38A1)	Cell Signaling	D9L2P	1:2000
SNAT2 (SLC38A2)	Abcam	ab90677	1:2000
SNAT4 (SLC38A4)	Abcam	ab58785	1:1000
SNAT5 (SLC38A5)	Abcam	ab72717	1:2000
LAT3 (SLC43A1)	Sigma	SAB4503399	1:1000
Sodium Potassium ATPase	Abcam	ab76020	1:5000
**Secondary antibodies**	**Source**	**Identifier**	**Dilution**
Goat anti-rabbit IgG, HRP-linked	Cell Signaling	7074	1:2000 – 1:10 000
Horse anti-mouse IgG, HRP-linked	Cell Signaling	7076	1:2000 – 1:4000

Table S13 Recovery of amino acids after repeated washing steps. Washing steps with 0.9% NaCl are shown as NaCl, washing steps with milli-Q water are shown as mQ. Multiplicators are shown before the washing step.

	0xNaCl/1xmQ		1xNaCl/1xmQ		2xNaCl/1xmQ	
	Mean	Std	Mean	Std	Mean	Std
F	892.9552	50.41358	748.3765	23.64324	716.0219	26.97582
L	1426.517	81.23733	1150.656	36.77618	1110.729	31.55748
I	1152.622	67.36913	929.9874	51.25624	879.2178	10.30266
M	254.8242	20.96939	228.97	11.57001	221.8048	10.53362
V	1208.482	82.74615	974.7722	49.55549	935.8306	29.04608
P	9520.033	425.6615	9230.468	545.921	8701.121	251.61
Y	992.7762	40.70558	812.5854	42.38067	773.854	26.2552
A	14874.3	738.834	13601.47	459.9651	13600.03	322.8966
T	4302.316	165.5492	4059.91	139.263	3946.179	63.26429
Q	289.0845	22.52361	255.3397	13.90706	224.3138	8.559583
G	28910.14	4325.78	19640.32	3345.344	19009.79	1047.712
E	22661.14	1805.414	21798.58	1146.214	20714.76	1156.223
S	1706.097	115.0672	1574.41	128.9708	1539.293	110.4684
D	16766.21	6431.221	14465.72	3370.373	13729.16	2399.64
K	766.659	70.63187	522.0725	74.62875	409.2678	22.32327
R	428.0397	65.99567	289.6747	26.96622	275.8929	26.87871
H	514.07	63.46167	361.0778	19.58092	358.7841	20.71313

Table S14. Parameters of amino acid quantification.

Analyte	Molecular formula	MW (g mol^{-1})	Parent ion (m/z)	CE (eV)	T_R (min)	Quantification	Base peak product ion (m/z)	Qualifier product ion (m/z)	LOD (ppm)	LLOQ (ppm)	Linear regression equation	Correlation coefficient	Ionisation polarity
Glycine	$C_2H_5NO_2$	75.07	76.0393	-	12.57	a	-	-	13.628	22.713	y=0.08387x +0.01813	0.99958	+
Alanine	$C_3H_7NO_2$	89.09	90.0550	-	11.95	a	-	-	15.02	25.034	y+0.07653 – 0.03334	0.99920	+
Valine	$C_5H_{11}NO_2$	117.15	118.0863	10.0	10.05	b	72.0813	72.0813	0.129	0.215	y=0.10335x +0.11064	0.99886	+
Leucine	$C_6H_{13}NO_2$	131.17	132.1019	10.0	8.98	b	86.0965	69.0704	0.161	0.2683	y=0.0953x +0.04235	0.99951	+
Isoleucine	$C_6H_{13}NO_2$	131.17	132.1019	10.0	8.57	b	86.0965	86.0965	0.2725	0.4541	y=0.09675x +0.05373	0.99916	+
Methionine	$C_5H_{11}NO_2S$	149.21	150.0583	13.5	9.53	b	56.0500	104.0530	0.1516	0.2526	y=0.09664x +0.02959	0.99975	+
Phenyl-alanine	$C_9H_{11}NO_2$	165.19	166.0865	12.0	8.48	b	120.0811	120.0811	0.037	0.0617	y=0.05395x +0.1147	0.99827	+
Proline	$C_5H_9NO_2$	115.13	116.0706	16.0	10.15	b	70.0654	70.0654	0.0387	0.0645	y=0.08993x +0.06677	0.99892	+
Serine	$C_3H_7NO_3$	105.09	106.0499	11.0	12.94	b	60.0448	60.0448	3.5664	5.944	y=0.09713x +0.22671	0.99778	+
Threonine	$C_4H_9NO_3$	119.12	120.0655	12.0	12.10	b	74.0603	74.0603	4.1761	6.9601	y=0.09751x +0.06334	0.99905	+
Tyrosine	$C_9H_{11}NO_3$	181.19	182.0812	12.5	11.00	b	136.0751	136.0751	1.9891	3.3151	y=0.10508x +0.08068	0.99806	+
Glutamine	$C_5H_{10}N_2O_3$	146.14	147.0764	13.0	12.48	b	84.0450	102.0548	4.4133	7.3554	y=0.09132x +0.11506	0.99604	+
Aspartic acid	$C_4H_7NO_4$	133.11	134.0448	-	13.30	a	-	-	32.093	53.488	y=0.09707x +0.00648	0.99952	+
Glutamic acid	$C_5H_9NO_4$	147.13	148.0604	12.0	12.75	b	84.0450	102.0547	14.753	24.588	y=0.09298x +0.05271	0.99908	+
Arginine	$C_6H_{14}N_4O_2$	174.20	175.1190	18.0	14.05	b	70.0658	158.0926	8.4227	14.038	y=0.10889x+ 0.02004	0.99974	+
Lysine	$C_6H_{14}N_2O_2$	146.19	147.1128	11.0	13.99	b	84.0808	101.1080	2.4048	4.008	y=0.09899x+ 0.03442	0.99935	+
Histidine	$C_6H_9N_3O_2$	155.15	156.0768	15.5	14.05	b	110.0710	110.0710	0.4415	0.7358	y=0.10406x+ 0.06246	0.99984	+

Table S15: Statistical analysis of Fig. 2 and 3 by One-way ANOVA with Tukey Kramer multiple comparison test. A q value> 4.656 equates to a p-value <0.05.

Fig. 2								
Comparison					Difference	q	Significance	p
Leucine								
Column	A	vs	Column	B	4.248	11.697	***	P<0.001
Column	A	vs	Column	C	21.423	58.996	***	P<0.001
Column	A	vs	Column	D	22.576	50.764	***	P<0.001
Column	A	vs	Column	E	2.566	7.066	***	P<0.001
Column	A	vs	Column	F	5.538	15.25	***	P<0.001
Column	A	vs	Column	G	1.006	2.642	ns	P>0.05
Column	A	vs	Column	H	18.455	50.824	***	P<0.001
Column	A	vs	Column	I	22.988	63.307	***	P<0.001
Column	A	vs	Column	J	22.911	66.509	***	P<0.001
Column	A	vs	Column	K	1.787	4.017	ns	P>0.05
Column	B	vs	Column	C	17.175	47.299	***	P<0.001
Column	B	vs	Column	D	18.329	41.214	***	P<0.001
Column	B	vs	Column	E	-1.682	4.631	ns	P>0.05
Column	B	vs	Column	F	1.29	3.553	ns	P>0.05
Column	B	vs	Column	G	-3.241	8.511	***	P<0.001
Column	B	vs	Column	H	14.208	39.126	***	P<0.001
Column	B	vs	Column	I	18.74	51.61	***	P<0.001
Column	B	vs	Column	J	18.664	54.179	***	P<0.001
Column	B	vs	Column	K	-2.461	5.533	**	P<0.01
Column	C	vs	Column	D	1.154	2.594	ns	P>0.05
Column	C	vs	Column	E	-18.857	51.93	***	P<0.001
Column	C	vs	Column	F	-15.885	43.746	***	P<0.001
Column	C	vs	Column	G	-20.416	53.608	***	P<0.001
Column	C	vs	Column	H	-2.968	8.172	***	P<0.001
Column	C	vs	Column	I	1.565	4.311	ns	P>0.05
Column	C	vs	Column	J	1.489	4.322	ns	P>0.05
Column	C	vs	Column	K	-19.636	44.153	***	P<0.001
Column	D	vs	Column	E	-20.011	44.995	***	P<0.001
Column	D	vs	Column	F	-17.039	38.313	***	P<0.001
Column	D	vs	Column	G	-21.57	46.961	***	P<0.001
Column	D	vs	Column	H	-4.121	9.267	***	P<0.001
Column	D	vs	Column	I	0.4116	0.9255	ns	P>0.05
Column	D	vs	Column	J	0.3349	0.7794	ns	P>0.05
Column	D	vs	Column	K	-20.79	40.484	***	P<0.001
Column	E	vs	Column	F	2.972	8.184	***	P<0.001
Column	E	vs	Column	G	-1.56	4.095	ns	P>0.05
Column	E	vs	Column	H	15.889	43.758	***	P<0.001
Column	E	vs	Column	I	20.422	56.241	***	P<0.001
Column	E	vs	Column	J	20.345	59.06	***	P<0.001
Column	E	vs	Column	K	-0.7792	1.752	ns	P>0.05
Column	F	vs	Column	G	-4.531	11.898	***	P<0.001
Column	F	vs	Column	H	12.918	35.574	***	P<0.001
Column	F	vs	Column	I	17.45	48.057	***	P<0.001
Column	F	vs	Column	J	17.374	50.434	***	P<0.001

Column	F	vs	Column	K	-3.751	8.434	***	P<0.001
Column	G	vs	Column	H	17.449	45.816	***	P<0.001
Column	G	vs	Column	I	21.982	57.718	***	P<0.001
Column	G	vs	Column	J	21.905	60.324	***	P<0.001
Column	G	vs	Column	K	0.7803	1.699	ns	P>0.05
Column	H	vs	Column	I	4.533	12.483	***	P<0.001
Column	H	vs	Column	J	4.456	12.936	***	P<0.001
Column	H	vs	Column	K	-16.668	37.48	***	P<0.001
Column	I	vs	Column	J	-0.07672	0.2227	ns	P>0.05
Column	I	vs	Column	K	-21.201	47.672	***	P<0.001
Column	J	vs	Column	K	-21.125	49.167	***	P<0.001
Glutamine								
Column	A	vs	Column	B	17.359	44.352	***	P<0.001
Column	A	vs	Column	C	15.983	44.044	***	P<0.001
Column	A	vs	Column	D	0.2762	0.7057	ns	P>0.05
Column	A	vs	Column	E	0.6196	1.649	ns	P>0.05
Column	A	vs	Column	F	5.013	12.184	***	P<0.001
Column	A	vs	Column	G	6.712	16.311	***	P<0.001
Column	A	vs	Column	H	-0.3667	1.091	ns	P>0.05
Column	A	vs	Column	I	2.713	7.899	***	P<0.001
Column	A	vs	Column	J	18.844	45.796	***	P<0.001
Column	B	vs	Column	C	-1.376	3.317	ns	P>0.05
Column	B	vs	Column	D	-17.083	38.834	***	P<0.001
Column	B	vs	Column	E	-16.739	39.302	***	P<0.001
Column	B	vs	Column	F	-12.346	26.964	***	P<0.001
Column	B	vs	Column	G	-10.647	23.255	***	P<0.001
Column	B	vs	Column	H	-17.726	45.289	***	P<0.001
Column	B	vs	Column	I	-14.646	36.808	***	P<0.001
Column	B	vs	Column	J	1.485	3.243	ns	P>0.05
Column	C	vs	Column	D	-15.707	37.873	***	P<0.001
Column	C	vs	Column	E	-15.364	38.42	***	P<0.001
Column	C	vs	Column	F	-10.97	25.292	***	P<0.001
Column	C	vs	Column	G	-9.272	21.376	***	P<0.001
Column	C	vs	Column	H	-16.35	45.055	***	P<0.001
Column	C	vs	Column	I	-13.27	35.875	***	P<0.001
Column	C	vs	Column	J	2.861	6.595	***	P<0.001
Column	D	vs	Column	E	0.3434	0.8062	ns	P>0.05
Column	D	vs	Column	F	4.737	10.346	***	P<0.001
Column	D	vs	Column	G	6.435	14.056	***	P<0.001
Column	D	vs	Column	H	-0.6429	1.642	ns	P>0.05
Column	D	vs	Column	I	2.437	6.125	**	P<0.01
Column	D	vs	Column	J	18.568	40.554	***	P<0.001
Column	E	vs	Column	F	4.394	9.886	***	P<0.001
Column	E	vs	Column	G	6.092	13.707	***	P<0.001
Column	E	vs	Column	H	-0.9863	2.626	ns	P>0.05
Column	E	vs	Column	I	2.094	5.475	**	P<0.01
Column	E	vs	Column	J	18.224	41.005	***	P<0.001
Column	F	vs	Column	G	1.698	3.574	ns	P>0.05
Column	F	vs	Column	H	-5.38	13.075	***	P<0.001

Column	F	vs	Column	I	-2.3	5.507	**	P<0.01
Column	F	vs	Column	J	13.831	29.109	***	P<0.001
Column	G	vs	Column	H	-7.078	17.202	***	P<0.001
Column	G	vs	Column	I	-3.998	9.573	***	P<0.001
Column	G	vs	Column	J	12.132	25.534	***	P<0.001
Column	H	vs	Column	I	3.08	8.966	***	P<0.001
Column	H	vs	Column	J	19.211	46.687	***	P<0.001
Column	I	vs	Column	J	16.131	38.621	***	P<0.001
Alanine								
Column	A	vs	Column	B	45.812	80.149	***	P<0.001
Column	A	vs	Column	C	43.569	76.226	***	P<0.001
Column	A	vs	Column	D	11.007	19.933	***	P<0.001
Column	A	vs	Column	E	7.669	11.619	***	P<0.001
Column	A	vs	Column	F	18.234	27.627	***	P<0.001
Column	A	vs	Column	G	2.148	3.759	ns	P>0.05
Column	A	vs	Column	H	7.78	14.52	***	P<0.001
Column	A	vs	Column	I	1.1	2.158	ns	P>0.05
Column	A	vs	Column	J	44.711	91.345	***	P<0.001
Column	B	vs	Column	C	-2.242	3.398	ns	P>0.05
Column	B	vs	Column	D	-34.804	54.104	***	P<0.001
Column	B	vs	Column	E	-38.143	51.691	***	P<0.001
Column	B	vs	Column	F	-27.578	37.373	***	P<0.001
Column	B	vs	Column	G	-43.663	66.156	***	P<0.001
Column	B	vs	Column	H	-38.032	60.436	***	P<0.001
Column	B	vs	Column	I	-44.712	73.647	***	P<0.001
Column	B	vs	Column	J	-1.101	1.865	ns	P>0.05
Column	C	vs	Column	D	-32.562	50.618	***	P<0.001
Column	C	vs	Column	E	-35.9	48.652	***	P<0.001
Column	C	vs	Column	F	-25.335	34.334	***	P<0.001
Column	C	vs	Column	G	-41.421	62.759	***	P<0.001
Column	C	vs	Column	H	-35.789	56.873	***	P<0.001
Column	C	vs	Column	I	-42.47	69.953	***	P<0.001
Column	C	vs	Column	J	1.141	1.933	ns	P>0.05
Column	D	vs	Column	E	-3.338	4.617	*	P<0.05
Column	D	vs	Column	F	7.227	9.995	***	P<0.001
Column	D	vs	Column	G	-8.859	13.771	***	P<0.001
Column	D	vs	Column	H	-3.227	5.276	*	P<0.05
Column	D	vs	Column	I	-9.908	16.824	***	P<0.001
Column	D	vs	Column	J	33.703	58.965	***	P<0.001
Column	E	vs	Column	F	10.565	13.07	***	P<0.001
Column	E	vs	Column	G	-5.521	7.481	***	P<0.001
Column	E	vs	Column	H	0.1111	0.1563	ns	P>0.05
Column	E	vs	Column	I	-6.569	9.507	***	P<0.001
Column	E	vs	Column	J	37.042	54.771	***	P<0.001
Column	F	vs	Column	G	-16.086	21.799	***	P<0.001
Column	F	vs	Column	H	-10.454	14.712	***	P<0.001
Column	F	vs	Column	I	-17.134	24.796	***	P<0.001
Column	F	vs	Column	J	26.477	39.149	***	P<0.001
Column	G	vs	Column	H	5.632	8.949	***	P<0.001

Column	G	vs	Column	I	-1.049	1.728	ns	P>0.05
Column	G	vs	Column	J	42.562	72.1	***	P<0.001
Column	H	vs	Column	I	-6.68	11.647	***	P<0.001
Column	H	vs	Column	J	36.931	66.449	***	P<0.001
Column	I	vs	Column	J	43.611	82.202	***	P<0.001
Proline								
Column	A	vs	Column	B	6.273	38.229	***	P<0.001
Column	A	vs	Column	C	4.163	25.372	***	P<0.001
Column	A	vs	Column	D	1.36	8.288	***	P<0.001
Column	A	vs	Column	E	-0.01167	0.0711	ns	P>0.05
Column	A	vs	Column	F	0.6367	3.88	ns	P>0.05
Column	A	vs	Column	G	4.756	32.861	***	P<0.001
Column	A	vs	Column	H	1.408	8.583	***	P<0.001
Column	B	vs	Column	C	-2.11	11.135	***	P<0.001
Column	B	vs	Column	D	-4.913	25.93	***	P<0.001
Column	B	vs	Column	E	-6.285	33.169	***	P<0.001
Column	B	vs	Column	F	-5.637	29.747	***	P<0.001
Column	B	vs	Column	G	-1.518	8.774	***	P<0.001
Column	B	vs	Column	H	-4.865	25.675	***	P<0.001
Column	C	vs	Column	D	-2.803	14.795	***	P<0.001
Column	C	vs	Column	E	-4.175	22.034	***	P<0.001
Column	C	vs	Column	F	-3.527	18.612	***	P<0.001
Column	C	vs	Column	G	0.5922	3.424	ns	P>0.05
Column	C	vs	Column	H	-2.755	14.54	***	P<0.001
Column	D	vs	Column	E	-1.372	7.239	***	P<0.001
Column	D	vs	Column	F	-0.7233	3.817	ns	P>0.05
Column	D	vs	Column	G	3.396	19.631	***	P<0.001
Column	D	vs	Column	H	0.04833	0.2551	ns	P>0.05
Column	E	vs	Column	F	0.6483	3.422	ns	P>0.05
Column	E	vs	Column	G	4.767	27.561	***	P<0.001
Column	E	vs	Column	H	1.42	7.494	***	P<0.001
Column	F	vs	Column	G	4.119	23.813	***	P<0.001
Column	F	vs	Column	H	0.7717	4.073	ns	P>0.05
Column	G	vs	Column	H	-3.347	19.352	***	P<0.001
Glycine								
Column	A	vs	Column	B	4.239	23.988	***	P<0.001
Column	A	vs	Column	C	3.079	15.504	***	P<0.001
Column	A	vs	Column	D	0.677	3.409	ns	P>0.05
Column	A	vs	Column	E	0.187	0.9415	ns	P>0.05
Column	A	vs	Column	F	0.2903	1.462	ns	P>0.05
Column	A	vs	Column	G	3.23	16.264	***	P<0.001
Column	A	vs	Column	H	0.7203	3.627	ns	P>0.05
Column	B	vs	Column	C	-1.16	5.722	**	P<0.01
Column	B	vs	Column	D	-3.562	17.573	***	P<0.001
Column	B	vs	Column	E	-4.052	19.99	***	P<0.001
Column	B	vs	Column	F	-3.949	19.48	***	P<0.001
Column	B	vs	Column	G	-1.009	4.977	*	P<0.05
Column	B	vs	Column	H	-3.519	17.359	***	P<0.001
Column	C	vs	Column	D	-2.402	10.818	***	P<0.001

Column	C	vs	Column	E	-2.892	13.025	***	P<0.001
Column	C	vs	Column	F	-2.789	12.559	***	P<0.001
Column	C	vs	Column	G	0.151	0.68	ns	P>0.05
Column	C	vs	Column	H	-2.359	10.623	***	P<0.001
Column	D	vs	Column	E	-0.49	2.207	ns	P>0.05
Column	D	vs	Column	F	-0.3867	1.741	ns	P>0.05
Column	D	vs	Column	G	2.553	11.498	***	P<0.001
Column	D	vs	Column	H	0.04333	0.1951	ns	P>0.05
Column	E	vs	Column	F	0.1033	0.4653	ns	P>0.05
Column	E	vs	Column	G	3.043	13.705	***	P<0.001
Column	E	vs	Column	H	0.5333	2.402	ns	P>0.05
Column	F	vs	Column	G	2.94	13.239	***	P<0.001
Column	F	vs	Column	H	0.43	1.936	ns	P>0.05
Column	G	vs	Column	H	-2.51	11.303	***	P<0.001
Glutamate								
Column	A	vs	Column	B	0.1067	2.258	ns	P>0.05
Column	A	vs	Column	C	0.4533	10.513	***	P<0.001
Column	A	vs	Column	D	0.8117	17.832	***	P<0.001
Column	A	vs	Column	E	0.3528	7.751	***	P<0.001
Column	B	vs	Column	C	0.3467	8.04	***	P<0.001
Column	B	vs	Column	D	0.705	15.488	***	P<0.001
Column	B	vs	Column	E	0.2461	5.408	**	P<0.01
Column	C	vs	Column	D	0.3583	8.691	***	P<0.001
Column	C	vs	Column	E	-0.1005	2.438	ns	P>0.05
Column	D	vs	Column	E	-0.4589	10.492	***	P<0.001
Arginine								
Column	A	vs	Column	B	1.022	5.077	**	P<0.01
Column	A	vs	Column	C	6.178	31.628	***	P<0.001
Column	A	vs	Column	D	2.271	11.627	***	P<0.001
Column	A	vs	Column	E	2.365	11.745	***	P<0.001
Column	B	vs	Column	C	5.156	25.608	***	P<0.001
Column	B	vs	Column	D	1.249	6.204	***	P<0.001
Column	B	vs	Column	E	1.343	6.48	***	P<0.001
Column	C	vs	Column	D	-3.907	20.001	***	P<0.001
Column	C	vs	Column	E	-3.813	18.94	***	P<0.001
Column	D	vs	Column	E	0.09347	0.4643	ns	P>0.05

Fig. 3								
Comparison					Difference	q	Significance	p
Leucine								
Column	A	vs	Column	B	3.315	4.516	*	P<0.05
Column	A	vs	Column	C	38.13	51.935	***	P<0.001
Column	A	vs	Column	D	25.699	35.003	***	P<0.001
Column	A	vs	Column	E	6.421	8.745	***	P<0.001
Column	A	vs	Column	F	8.383	11.418	***	P<0.001
Column	A	vs	Column	G	5.304	7.224	***	P<0.001
Column	B	vs	Column	C	34.815	47.419	***	P<0.001
Column	B	vs	Column	D	22.384	30.488	***	P<0.001
Column	B	vs	Column	E	3.106	4.23	ns	P>0.05

Column	B	vs	Column	F	5.068	6.903	***	P<0.001
Column	B	vs	Column	G	1.988	2.708	ns	P>0.05
Column	C	vs	Column	D	-12.431	16.932	***	P<0.001
Column	C	vs	Column	E	-31.709	43.189	***	P<0.001
Column	C	vs	Column	F	-29.747	40.516	***	P<0.001
Column	C	vs	Column	G	-32.826	44.711	***	P<0.001
Column	D	vs	Column	E	-19.278	26.258	***	P<0.001
Column	D	vs	Column	F	-17.316	23.585	***	P<0.001
Column	D	vs	Column	G	-20.395	27.78	***	P<0.001
Column	E	vs	Column	F	1.962	2.673	ns	P>0.05
Column	E	vs	Column	G	-1.117	1.522	ns	P>0.05
Column	F	vs	Column	G	-3.08	4.195	ns	P>0.05
Glutamine								
Column	A	vs	Column	B	30.356	31.521	***	P<0.001
Column	A	vs	Column	C	26.263	28.11	***	P<0.001
Column	A	vs	Column	D	3.201	3.427	ns	P>0.05
Column	A	vs	Column	E	10	10.703	***	P<0.001
Column	A	vs	Column	F	5.11	5.469	**	P<0.01
Column	A	vs	Column	G	0.6032	0.6456	ns	P>0.05
Column	B	vs	Column	C	-4.094	4.251	ns	P>0.05
Column	B	vs	Column	D	-27.155	28.197	***	P<0.001
Column	B	vs	Column	E	-20.357	21.138	***	P<0.001
Column	B	vs	Column	F	-25.246	26.215	***	P<0.001
Column	B	vs	Column	G	-29.753	30.895	***	P<0.001
Column	C	vs	Column	D	-23.061	24.683	***	P<0.001
Column	C	vs	Column	E	-16.263	17.407	***	P<0.001
Column	C	vs	Column	F	-21.153	22.64	***	P<0.001
Column	C	vs	Column	G	-25.66	27.464	***	P<0.001
Column	D	vs	Column	E	6.798	7.276	***	P<0.001
Column	D	vs	Column	F	1.909	2.043	ns	P>0.05
Column	D	vs	Column	G	-2.598	2.781	ns	P>0.05
Column	E	vs	Column	F	-4.889	5.233	**	P<0.01
Column	E	vs	Column	G	-9.396	10.057	***	P<0.001
Column	F	vs	Column	G	-4.507	4.824	*	P<0.05
Alanine								
Column	A	vs	Column	B	65.213	45.967	***	P<0.001
Column	A	vs	Column	C	59.771	45.041	***	P<0.001
Column	A	vs	Column	D	5.202	3.92	ns	P>0.05
Column	A	vs	Column	E	11.305	8.519	***	P<0.001
Column	A	vs	Column	F	10.302	7.763	***	P<0.001
Column	A	vs	Column	G	1.263	0.9518	ns	P>0.05
Column	B	vs	Column	C	-5.441	3.835	ns	P>0.05
Column	B	vs	Column	D	-60.01	42.3	***	P<0.001
Column	B	vs	Column	E	-53.907	37.998	***	P<0.001
Column	B	vs	Column	F	-54.911	38.706	***	P<0.001
Column	B	vs	Column	G	-63.95	45.077	***	P<0.001
Column	C	vs	Column	D	-54.569	41.121	***	P<0.001
Column	C	vs	Column	E	-48.466	36.522	***	P<0.001
Column	C	vs	Column	F	-49.47	37.278	***	P<0.001

Column	C	vs	Column	G	-58.508	44.089	***	P<0.001
Column	D	vs	Column	E	6.103	4.599	*	P<0.05
Column	D	vs	Column	F	5.099	3.843	ns	P>0.05
Column	D	vs	Column	G	-3.939	2.969	ns	P>0.05
Column	E	vs	Column	F	-1.004	0.7564	ns	P>0.05
Column	E	vs	Column	G	-10.042	7.567	***	P<0.001
Column	F	vs	Column	G	-9.039	6.811	***	P<0.001
Proline								
Column	A	vs	Column	B	12.006	16.37	***	P<0.001
Column	A	vs	Column	C	10.449	14.686	***	P<0.001
Column	A	vs	Column	D	2.229	3.039	ns	P>0.05
Column	A	vs	Column	E	3.327	4.676	*	P<0.05
Column	A	vs	Column	F	10.763	13.53	***	P<0.001
Column	A	vs	Column	G	5.051	7.099	***	P<0.001
Column	B	vs	Column	C	-1.557	2.122	ns	P>0.05
Column	B	vs	Column	D	-9.777	12.956	***	P<0.001
Column	B	vs	Column	E	-8.679	11.834	***	P<0.001
Column	B	vs	Column	F	-1.243	1.525	ns	P>0.05
Column	B	vs	Column	G	-6.955	9.483	***	P<0.001
Column	C	vs	Column	D	-8.221	11.209	***	P<0.001
Column	C	vs	Column	E	-7.123	10.01	***	P<0.001
Column	C	vs	Column	F	0.3133	0.3939	ns	P>0.05
Column	C	vs	Column	G	-5.399	7.588	***	P<0.001
Column	D	vs	Column	E	1.098	1.497	ns	P>0.05
Column	D	vs	Column	F	8.534	10.469	***	P<0.001
Column	D	vs	Column	G	2.822	3.848	ns	P>0.05
Column	E	vs	Column	F	7.436	9.347	***	P<0.001
Column	E	vs	Column	G	1.724	2.423	ns	P>0.05
Column	F	vs	Column	G	-5.712	7.18	***	P<0.001
Glycine								
Column	A	vs	Column	B	2.116	18.266	***	P<0.001
Column	A	vs	Column	C	0.1239	1.07	ns	P>0.05
Column	A	vs	Column	D	-0.1208	1.043	ns	P>0.05
Column	B	vs	Column	C	-1.992	17.196	***	P<0.001
Column	B	vs	Column	D	-2.237	19.309	***	P<0.001
Column	C	vs	Column	D	-0.2447	2.113	ns	P>0.05
Glutamate								
Column	A	vs	Column	B	1.275	5.497	**	P<0.01
Column	A	vs	Column	C	4.668	20.135	***	P<0.001
Column	A	vs	Column	D	-0.9223	3.859	ns	P>0.05
Column	A	vs	Column	E	-0.1427	0.5201	ns	P>0.05
Column	A	vs	Column	F	-0.2483	1.039	ns	P>0.05
Column	B	vs	Column	C	3.394	14.638	***	P<0.001
Column	B	vs	Column	D	-2.197	9.192	***	P<0.001
Column	B	vs	Column	E	-1.417	5.166	**	P<0.01
Column	B	vs	Column	F	-1.523	6.372	***	P<0.001
Column	C	vs	Column	D	-5.591	23.393	***	P<0.001
Column	C	vs	Column	E	-4.811	17.538	***	P<0.001
Column	C	vs	Column	F	-4.917	20.573	***	P<0.001

Column	D	vs	Column	E	0.7796	2.781	ns	P>0.05
Column	D	vs	Column	F	0.674	2.741	ns	P>0.05
Column	E	vs	Column	F	-0.1056	0.3766	ns	P>0.05
Arginine								
Column	A	vs	Column	B	-0.8931	1.264	ns	P>0.05
Column	A	vs	Column	C	15.693	22.209	***	P<0.001
Column	A	vs	Column	D	0.4964	0.6571	ns	P>0.05
Column	A	vs	Column	E	8.861	12.541	***	P<0.001
Column	B	vs	Column	C	16.586	23.473	***	P<0.001
Column	B	vs	Column	D	1.389	1.839	ns	P>0.05
Column	B	vs	Column	E	9.754	13.805	***	P<0.001
Column	C	vs	Column	D	-15.196	20.117	***	P<0.001
Column	C	vs	Column	E	-6.831	9.668	***	P<0.001
Column	D	vs	Column	E	8.365	11.074	***	P<0.001

References:

1. Arriza JL, Fairman WA, Wadiche JI, Murdoch GH, Kavanaugh MP, Amara SG. Functional comparisons of three glutamate transporter subtypes cloned from human motor cortex. *J Neurosci* **14**, 5559-5569 (1994).

2. Kanai Y, Nussberger S, Romero MF, Boron WF, Hebert SC, Hediger MA. Electrogenic properties of the epithelial and neuronal high affinity glutamate transporter. *J Biol Chem* **270**, 16561-16568 (1995).

3. Watzke N, Rauen T, Bamberg E, Grewer C. On the mechanism of proton transport by the neuronal excitatory amino acid carrier 1. *J Gen Physiol* **116**, 609-622. (2000).

4. Wang J, Zielewicz L, Grewer C. A K(+)/Na(+) co-binding state: Simultaneous versus competitive binding of K(+) and Na(+) to glutamate transporters. *J Biol Chem* **294**, 12180-12190 (2019).

5. Arriza JL*, et al.* Cloning and expression of a human neutral amino acid transporter with structural similarity to the glutamate transporter gene family. *J Biol Chem* **268**, 15329-15332 (1993).

6. Utsunomiya-Tate N, Endou H, Kanai Y. Cloning and functional characterization of a system ASC-like Na+-dependent neutral amino acid transporter. *J Biol Chem* **271**, 14883-14890. (1996).

7. Scalise M, Pochini L, Panni S, Pingitore P, Hedfalk K, Indiveri C. Transport mechanism and regulatory properties of the human amino acid transporter ASCT2 (SLC1A5). *Amino Acids* **46**, 2463-2475 (2014).

8. Fairman WA, Vandenberg RJ, Arriza JL, Kavanaugh MP, Amara SG. An excitatory amino-acid transporter with properties of a ligand-gated chloride channel. *Nature* **375**, 599-603 (1995).

9. Arriza JL, Eliasof S, Kavanaugh MP, Amara SG. Excitatory amino acid transporter 5, a retinal glutamate transporter coupled to a chloride conductance. *Proc Natl Acad Sci U S A* **94**, 4155-4160 (1997).

10. Liu QR, Lopez-Corcuera B, Mandiyan S, Nelson H, Nelson N. Cloning and expression of a spinal cord- and brain-specific glycine transporter with novel structural features. *J Biol Chem* **268**, 22802-22808 (1993).

11. Lopez-Corcuera B, Martinez-Maza R, Nunez E, Roux M, Supplisson S, Aragon C. Differential properties of two stably expressed brain-specific glycine transporters. *J Neurochem* **71**, 2211-2219 (1998).

12. Ramamoorthy S*, et al.* Functional characterization and chromosomal localization of a cloned taurine transporter from human placenta. *Biochem J* **300**, 893-900. (1994).

13. Shafqat S*, et al.* Human brain-specific L-proline transporter: molecular cloning, functional expression, and chromosomal localization of the gene in human and mouse genomes. *Mol Pharmacol* **48**, 219-229 (1995).

14. Sloan JL, Mager S. Cloning and functional expression of a human Na(+) and Cl(-)-dependent neutral and cationic amino acid transporter B(0+). *J Biol Chem* **274**, 23740-23745 (1999).

15. Broer A*, et al.* The orphan transporter v7-3 (slc6a15) is a Na+-dependent neutral amino acid transporter (B0AT2). *Biochem J* **393**, 421-430 (2006).

16. Takanaga H, Mackenzie B, Peng JB, Hediger MA. Characterization of a branched-chain amino-acid transporter SBAT1 (SLC6A15) that is expressed in human brain. *Biochem Biophys Res Commun* **337**, 892-900 (2005).

17. Zaia KA, Reimer RJ. Synaptic Vesicle Protein NTT4/XT1 (SLC6A17) Catalyzes Na+-coupled Neutral Amino Acid Transport. *J Biol Chem* **284**, 8439-8448 (2009).

18. Bohmer C*, et al.* Characterization of mouse amino acid transporter B0AT1 (slc6a19). *Biochem J* **389**, 745-751 (2005).

19. Cheng Q*, et al.* Identification of novel inhibitors of the amino acid transporter B(0) AT1 (SLC6A19), a potential target to induce protein restriction and to treat type 2 diabetes. *Br J Pharmacol* **174**, 468-482 (2017).

20. Kowalczuk S, Broer A, Munzinger M, Tietze N, Klingel K, Broer S. Molecular cloning of the mouse IMINO system: an Na+- and Cl--dependent proline transporter. *Biochemical Journal* **386**, 417-422 (2005).

21. Takanaga H, Mackenzie B, Suzuki Y, Hediger MA. Identification of Mammalian Proline Transporter SIT1 (SLC6A20) with Characteristics of Classical System Imino. *J Biol Chem* **280**, 8974-8984 (2005).

22. Wang H, Kavanaugh MP, North RA, Kabat D. Cell-surface receptor for ecotropic murine retroviruses is a basic amino-acid transporter. *Nature* **352**, 729-731 (1991).

23. Kavanaugh MP*, et al.* Control of cationic amino acid transport and retroviral receptor functions in a membrane protein family. *J Biol Chem* **269**, 15445-15450 (1994).

24. Hosokawa H, Sawamura T, Kobayashi S, Ninomiya H, Miwa S, Masaki T. Cloning and characterization of a brain-specific cationic amino acid transporter. *J Biol Chem* **272**, 8717-8722 (1997).

25. Yanagida O*, et al.* Human L-type amino acid transporter 1 (LAT1): characterization of function and expression in tumor cell lines. *Biochim Biophys Acta* **1514**, 291-302. (2001).

26. Meier C, Ristic Z, Klauser S, Verrey F. Activation of system L heterodimeric amino acid exchangers by intracellular substrates. *Embo J* **21**, 580-589 (2002).

27. Broer A, Wagner CA, Lang F, Broer S. The heterodimeric amino acid transporter 4F2hc/y+LAT2 mediates arginine efflux in exchange with glutamine. *Biochem J* **349 Pt 3**, 787-795 (2000).

28. Chubb S, Kingsland AL, Broer A, Broer S. Mutation of the 4F2 heavy-chain carboxy terminus causes y+ LAT2 light-chain dysfunction. *Mol Membr Biol* **23**, 255-267 (2006).

29. Pfeiffer R, Rossier G, Spindler B, Meier C, Kuhn L, Verrey F. Amino acid transport of y+L-type by heterodimers of 4F2hc/CD98 and members of the glycoprotein-associated amino acid transporter family. *Embo J* **18**, 49-57 (1999).

30. Kanai Y*, et al.* Transport properties of a system y+L neutral and basic amino acid transporter. Insights into the mechanisms of substrate recognition. *J Biol Chem* **275**, 20787-20793. (2000).

31. Segawa H, Fukasawa Y, Miyamoto K, Takeda E, Endou H, Kanai Y. Identification and functional characterization of a Na+-independent neutral amino acid transporter with broad substrate selectivity. *J Biol Chem* **274**, 19745-19751 (1999).

32. Mizoguchi K*, et al.* Human cystinuria-related transporter: localization and functional characterization. *Kidney Int* **59**, 1821-1833. (2001).

33. Torras-Llort M*, et al.* Sequential amino acid exchange across b(0,+)-like system in chicken brush border jejunum. *J Membr Biol* **180**, 213-220. (2001).

34. Fukasawa Y*, et al.* Identification and characterization of a Na(+)-independent neutral amino acid transporter that associates with the 4F2 heavy chain and exhibits substrate selectivity for small neutral D- and L-amino acids. *J Biol Chem* **275**, 9690-9698. (2000).

35. Bassi MT*, et al.* Identification and characterisation of human xCT that co-expresses, with 4F2 heavy chain, the amino acid transport activity system xc. *Pflugers Arch* **442**, 286-296. (2001).

36. Matsuo H*, et al.* Identification of a novel Na+-independent acidic amino acid transporter with structural similarity to the member of a heterodimeric amino acid transporter family associated with unknown heavy chains. *J Biol Chem* **277**, 21017-21026 (2002).

37. Nagamori S*, et al.* Novel cystine transporter in renal proximal tubule identified as a missing partner of cystinuria-related plasma membrane protein rBAT/SLC3A1. *Proc Natl Acad Sci U S A* **113**, 775-780 (2016).

38. Kim DK, Kanai Y, Chairoungdua A, Matsuo H, Cha SH, Endou H. Expression cloning of a Na+-independent aromatic amino acid transporter with structural similarity to H+/monocarboxylate transporters. *J Biol Chem* **276**, 17221-17228 (2001).

39. Boll M, Foltz M, Rubio-Aliaga I, Kottra G, Daniel H. Functional characterization of two novel mammalian electrogenic proton-dependent amino acid cotransporters. *J Biol Chem* **277**, 22966-22973 (2002).

40. Pillai SM, Meredith D. SLC36A4 (hPAT4) Is a High Affinity Amino Acid Transporter When Expressed in Xenopus laevis Oocytes. *J Biol Chem* **286**, 2455-2460 (2011).

41. Albers A*, et al.* Na+ transport by the neural glutamine transporter ATA1. *Pflugers Archiv-European Journal of Physiology* **443**, 92-101 (2001).

42. Mackenzie B, Schafer MK, Erickson JD, Hediger MA, Weihe E, Varoqui H. Functional properties and cellular distribution of the system A glutamine transporter SNAT1 support specialized roles in central neurons. *J Biol Chem* **278**, 23720-23730 (2003).

43. Yao D*, et al.* A novel system A isoform mediating Na+/neutral amino acid cotransport. *J Biol Chem* **275**, 22790-22797 (2000).

44. Fei YJ*, et al.* Primary structure, genomic organization, and functional and electrogenic characteristics of human system N 1, a Na+- and H+-coupled glutamine transporter. *J Biol Chem* **275**, 23707-23717 (2000).

45. Broer A*, et al.* Regulation of the glutamine transporter SN1 by extracellular pH and intracellular sodium ions. *J Physiol* **539**, 3-14 (2002).

46. Sugawara M*, et al.* Structure and function of ATA3, a new subtype of amino acid transport system A, primarily expressed in the liver and skeletal muscle. *Biochim Biophys Acta* **1509**, 7-13 (2000).

47. Hatanaka T*, et al.* Evidence for the transport of neutral as well as cationic amino acids by ATA3, a novel and liver-specific subtype of amino acid transport system A. *Biochim Biophys Acta* **1510**, 10-17 (2001).

48. Nakanishi T*, et al.* Structure, function, and tissue expression pattern of human SN2, a subtype of the amino acid transport system N. *Biochem Biophys Res Commun* **281**, 1343-1348 (2001).

49. Babu E*, et al.* Identification of a novel system L amino acid transporter structurally distinct from heterodimeric amino acid transporters. *J Biol Chem* **278**, 43838-43845 (2003).

50. Bodoy S, Martin L, Zorzano A, Palacin M, Estevez R, Bertran J. Identification of LAT4, a novel amino acid transporter with system L activity. *J Biol Chem* **280**, 12002-12011 (2005).
