# Supplementary material for: Quantitative modelling of amino acid transport and homeostasis in mammalian cells
Source: Nat Commun. 2021 Sep 6;12:5282. doi: 10.1038/s41467-021-25563-x (PMC8421413; doi:10.1038/s41467-021-25563-x)
Supplement: Supplementary file 3 — Reporting Summary [file 41467_2021_25563_MOESM3_ESM.pdf]

nature research

Corresponding author(s): Stefan Broer
Last updated by author(s): 27/07/2021

# Reporting Summary

Nature Research wishes to improve the reproducibility of the work that we publish. This form provides structure for consistency and transparency in reporting. For further information on Nature Research policies, see our Editorial Policies and the Editorial Policy Checklist.

## Statistics

For all statistical analyses, confirm that the following items are present in the figure legend, table legend, main text, or Methods section.

| n/a | Confirmed | |
|---|---|---|
| ☐ | ☒ | The exact sample size (*n*) for each experimental group/condition, given as a discrete number and unit of measurement |
| ☐ | ☒ | A statement on whether measurements were taken from distinct samples or whether the same sample was measured repeatedly |
| ☐ | ☒ | The statistical test(s) used AND whether they are one- or two-sided<br>*Only common tests should be described solely by name; describe more complex techniques in the Methods section.* |
| ☐ | ☒ | A description of all covariates tested |
| ☐ | ☒ | A description of any assumptions or corrections, such as tests of normality and adjustment for multiple comparisons |
| ☐ | ☒ | A full description of the statistical parameters including central tendency (e.g. means) or other basic estimates (e.g. regression coefficient) AND variation (e.g. standard deviation) or associated estimates of uncertainty (e.g. confidence intervals) |
| ☐ | ☒ | For null hypothesis testing, the test statistic (e.g. $F$, $t$, $r$) with confidence intervals, effect sizes, degrees of freedom and $P$ value noted<br>*Give P values as exact values whenever suitable.* |
| ☒ | ☐ | For Bayesian analysis, information on the choice of priors and Markov chain Monte Carlo settings |
| ☐ | ☒ | For hierarchical and complex designs, identification of the appropriate level for tests and full reporting of outcomes |
| ☐ | ☒ | Estimates of effect sizes (e.g. Cohen's *d*, Pearson's *r*), indicating how they were calculated |

*Our web collection on statistics for biologists contains articles on many of the points above.*

## Software and code

Policy information about availability of computer code

| | |
|---|---|
| Data collection | Custom Matlab script (Matlab R2019b), NIH Image J, Mass spectrometer software Xcalibur 4.1, TraceFinder 3.3, DEXSI 1.11, Oncomine (www.oncomine.org) |
| Data analysis | Origin 2020, Excel 2019, Graphpad prism 9, Custom Matlab script (available through Github JDFC_v1.1, for Matlab 2019 https://github.com/stefanbroeer/JDFC_v1.1) |

For manuscripts utilizing custom algorithms or software that are central to the research but not yet described in published literature, software must be made available to editors and reviewers. We strongly encourage code deposition in a community repository (e.g. GitHub). See the Nature Research guidelines for submitting code & software for further information.

## Data

Policy information about availability of data

All manuscripts must include a data availability statement. This statement should provide the following information, where applicable:
- Accession codes, unique identifiers, or web links for publicly available datasets
- A list of figures that have associated raw data
- A description of any restrictions on data availability

Source data for this manuscript are available as an Excel source data file associated with the manuscript. Figure files and source data files can be found: Bröer, Stefan (2021), "AA Homeostasis", Mendeley Data, V1, doi: 10.17632/6k3k4hcftt.1

# Field-specific reporting

Please select the one below that is the best fit for your research. If you are not sure, read the appropriate sections before making your selection.

☒ Life sciences ☐ Behavioural & social sciences ☐ Ecological, evolutionary & environmental sciences

For a reference copy of the document with all sections, see nature.com/documents/nr-reporting-summary-flat.pdf

# Life sciences study design

All studies must disclose on these points even when the disclosure is negative.

| | |
|---|---|
| Sample size | Sample sizes was determined using the equation: N≈2SD2(zα+zβ)/Δ2 where N is the number of samples required, SD the typical experimental standard deviation, zα is the chance of a type I error (0.05) and zβ is the chance of a type II error (0.1), both as a fraction of 1. Δ is the difference that the experiment should be able to detect. |
| Data exclusions | No datasets were excluded from analysis |
| Replication | All experiments were reproducible and were included in the datasets shown in the manuscript. The number of independent samples are given in the figure legends. A minimum of 3 experimental repeats was performed. |
| Randomization | Samples were not randomized. The risk of introducing experimental errors by randomizing samples in complex experiments is very high. |
| Blinding | Analysis was not blinded. Readouts and results in all experiments were numerical data that were used for calculations to determine transport rates and amino acid concentrations. Data analysis requires specific expertise that cannot be readily transferred to a blinded observer. |

# Reporting for specific materials, systems and methods

We require information from authors about some types of materials, experimental systems and methods used in many studies. Here, indicate whether each material, system or method listed is relevant to your study. If you are not sure if a list item applies to your research, read the appropriate section before selecting a response.

## Materials & experimental systems

| n/a | Involved in the study | |
|---|---|---|
| ☐ | ☒ | Antibodies |
| ☐ | ☒ | Eukaryotic cell lines |
| ☒ | ☐ | Palaeontology and archaeology |
| ☒ | ☐ | Animals and other organisms |
| ☒ | ☐ | Human research participants |
| ☒ | ☐ | Clinical data |
| ☒ | ☐ | Dual use research of concern |

## Methods

| n/a | Involved in the study | |
|---|---|---|
| ☒ | ☐ | ChIP-seq |
| ☒ | ☐ | Flow cytometry |
| ☒ | ☐ | MRI-based neuroimaging |

## Antibodies

Antibodies used

EAAT3 (SLC1A1) Gifted by David Pow
EAAT1 (SLC1A3) Abcam ab416
ASCT1 (SLC1A4) Cell Signaling 8442
ASCT2 (SLC1A5) Cell Signaling D7C12
GlyT2 (SLC6A9) Abcam ab99098
B0AT2 (SLC6A15) Pineda Antibody Service Custom antibody
CAT1 (SLC7A1) MyBioSource MBS176763
LAT1 (SLC7A5) Cell Signaling 5347
y+LAT2 (SLC7A6) Abcam ab235054
LAT2 (SLC7A8) Abcam ab123896
xCT (SLC7A11) Abcam ab175186
SNAT1 (SLC38A1) Cell Signaling D9L2P
SNAT2 (SLC38A2) Abcam ab90677
SNAT4 (SLC38A4) Abcam ab58785
SNAT5 (SLC38A5) Abcam ab72717
LAT3 (SLC43A1) Sigma SAB4503399
Sodium Potassium ATPase Abcam ab76020
Secondary antibodies Source Identifier
Goat anti-rabbit IgG, HRP-linked Cell Signaling 7074
Horse anti-mouse IgG, HRP-linked Cell Signaling 7076

Validation

EAAT3 (SLC1A1) Braidy N, Alicajic H, Pow D, Smith J, Jugder BE, Brew BJ, Nicolazzo JA, Guillemin GJ. Potential Mechanism of Cellular Uptake of the Excitotoxin Quinolinic Acid in Primary Human Neurons. Mol Neurobiol. 2021 Jan;58(1):34-54. doi: 10.1007/s12035-020-02046-6. Epub 2020 Sep 6. PMID: 32894500.
EAAT1 (SLC1A3) Abcam 416 Validated on mouse brain synaptosomes and tissue lysate by western blotting in house and by western blots provided by Manufacturer
ASCT1 (SLC1A4) Validated by western blotting and silencing: Bröer A, Gauthier-Coles G, Rahimi F, et al. Ablation of the ASCT2 (SLC1A5) gene encoding a neutral amino acid transporter reveals transporter plasticity and redundancy in cancer cells. J Biol Chem. 2019;294(11):4012-4026. doi:10.1074/jbc.RA118.006378
ASCT2 (SLC1A5) Validated by western blotting and CrispR ko: Bröer A, Rahimi F, Bröer S. Deletion of Amino Acid Transporter ASCT2 (SLC1A5) Reveals an Essential Role for Transporters SNAT1 (SLC38A1) and SNAT2 (SLC38A2) to Sustain Glutaminolysis in Cancer Cells. J Biol Chem. 2016;291(25):13194-13205. doi:10.1074/jbc.M115.700534
GlyT2 (SLC6A9) Abcam ab99098 Western blots on Panc1 cells as provided by the manufacturer
B0AT2 (SLC6A15) Pineda Antibody Service Custom antibody, Western blot on mouse brain synaptosomes
CAT1 (SLC7A1) MyBioSource MBS176763 Western blot on Placenta and 3 cell lines as provided by the manufacturer
LAT1 (SLC7A5) Cell Signaling 5347 Validated by western blot on cell lines: Bröer A, Gauthier-Coles G, Rahimi F, et al. Ablation of the ASCT2 (SLC1A5) gene encoding a neutral amino acid transporter reveals transporter plasticity and redundancy in cancer cells. J Biol Chem. 2019;294(11):4012-4026. doi:10.1074/jbc.RA118.006378
y+LAT2 (SLC7A6) Abcam ab235054 Validated by Western blotting on mouse heart tissue and immunofluorscence on sections of human small intestine as provided by manufacturer.
LAT2 (SLC7A8) Abcam ab123896 Validated by transient transfection and western blotting in HEK293 cells.
xCT (SLC7A11) Abcam ab175186 Validated by western blotting on three tissues and two cell lines as provided by manufacturer
SNAT1 (SLC38A1) Cell Signaling D9L2P Validated by western blotting and silencing Bröer A, Rahimi F, Bröer S. Deletion of Amino Acid Transporter ASCT2 (SLC1A5) Reveals an Essential Role for Transporters SNAT1 (SLC38A1) and SNAT2 (SLC38A2) to Sustain Glutaminolysis in Cancer Cells. J Biol Chem. 2016;291(25):13194-13205. doi:10.1074/jbc.M115.700534
SNAT2 (SLC38A2) Abcam ab90677 Validated by western blotting and silencing Bröer A, Rahimi F, Bröer S. Deletion of Amino Acid Transporter ASCT2 (SLC1A5) Reveals an Essential Role for Transporters SNAT1 (SLC38A1) and SNAT2 (SLC38A2) to Sustain Glutaminolysis in Cancer Cells. J Biol Chem. 2016;291(25):13194-13205. doi:10.1074/jbc.M115.700534
SNAT4 (SLC38A4) Abcam ab58785 validated by western blotting on Jurkat cells as provided by the manufacturer
SNAT5 (SLC38A5) Abcam ab72717 validated by western blotting in tissue lysate and immunohistochemistry as provided by the manusfacturer
LAT3 (SLC43A1) Sigma SAB4503399 validated by western blotting on Jurkat and A549 cells provided by the manufacturer
Sodium Potassium ATPase Abcam ab76020 Validated by Western blotting on multiple cell lines Bröer A, Gauthier-Coles G, Rahimi F, et al. Ablation of the ASCT2 (SLC1A5) gene encoding a neutral amino acid transporter reveals transporter plasticity and redundancy in cancer cells. J Biol Chem. 2019;294(11):4012-4026. doi:10.1074/jbc.RA118.006378
Secondary antibodies Source Identifier
Goat anti-rabbit IgG, HRP-linked Cell Signaling 7074 validation in conjunction with validation of primary antibodies
Horse anti-mouse IgG, HRP-linked Cell Signaling 7076 validation in conjunction with primary antibodies

## Eukaryotic cell lines

Policy information about cell lines

Cell line source(s)

Cell lines (U87-MG and A549) were obtained through ATCC

Authentication

Cell lines were not authenticated

Mycoplasma contamination

Cell lines tested negative for mycoplasma contamination

Commonly misidentified lines
(See ICLAC register)

Not commonly misidentified cell lines were used in this study
